# Synergized Tri‐Phase Anode via Purposive Ba^2+^ Doping at the La^3+^ Site in LaFe_0.7_Ni_0.3_O_3‐δ_ for Improved SOFC Performance

**DOI:** 10.1002/advs.75578

**Published:** 2026-05-08

**Authors:** Qian Yang, Shuaihang Chen, Yongli Peng, Xuelong Li, Xiaojian Pan, Fang Wang, Nanya Long, Li Li, Yilin Wang, Lina Su, Yuesong Shen, Ling Huang

**Affiliations:** ^1^ State Key Laboratory of Chemistry and Utilization of Carbon Based Energy Resources College of Chemistry Xinjiang University Urumqi China; ^2^ School of Materials Science and Engineering Nanjing Tech University Nanjing China

**Keywords:** anode, Ba^2+^ doping, HOR, solid oxide fuel cell, tri‐phase

## Abstract

Although perovskites are promising anode materials for solid oxide fuel cells (SOFCs), poor hydrogen oxidation reaction (HOR) kinetics and low peak power density (PPD) are usually limited by the single‐phase composition. Herein, purposive doping of Ba^2+^ at the La^3+^ site of La_0.5_Ba_0.5_Fe_0.7_Ni_0.3_O_3‐δ_ under reducing atmosphere triggers the formation of Ruddlesden‐Popper perovskite (La_2_Ba)Fe_2_O_7_, hexagonal Ba_6_La_2_Fe_4_O_15_, and exsolved cubic Ni_3_Fe nanoparticles, which facilitates excellent ionic and electronic conductivity, extra oxygen vacancies, and accelerated HOR kinetics. The as‐fabricated SOFC containing a tri‐phase anode made from the above composite leads to 57% polarization resistance reduction (from 0.28 to 0.12 Ω cm^2^), doubled PPD with a record value of 1.25 W cm^−2^ at 800 °C of that without Ba^2+^ doping, negligible voltage decrease rate of ∼8.9 × 10^−5^·V h^−1^, and 300‐h durability operated at 750°C.

## Graphical abstract

Purposive doping of low electronegative Ba^2^^+^ (0.89) at La^3^^+^ (1.1) site in LaFe_0.7_Ni_0.3_O_3‐δ_ induces the formation of a tri‐phase composite, which shortens Fe─O bond and lowers activation energy, thereby significantly improves electrical conductivity and catalytic activity, and finally offers a record‐high peak power density in La_0.5_Ba_0.5_Fe_0.7_Ni_0.3_O_3‐δ_‐based SOFC.

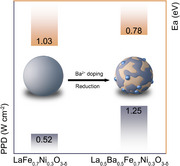

## 1 Introduction

Solid oxide fuel cells (SOFCs) that directly convert a wide variety of chemical fuels such as H_2_, CH_4_, CH_3_OH, and CO to electricity with much higher efficiency than current thermal power plants or combustion engines have drawn particular attention in satisfying the rapidly increased electricity demand raised by the fast‐growing usage of artificial intelligence, big data servers, and electric vehicles [1, 2, 3]. As one of the key components in SOFCs, an ideal anode should possess perfect electronic/ionic conductivity for rapid charge carrier transport, sufficient electro/catalytic activity for quick fuel oxidation reactions, and excellent structural stability for long‐term and high‐temperature operation [4, 5, 6, 7, 8].

Perovskite oxides are considered promising anode materials due to their high mixed ionic‐electronic conductivity (MIEC), inherent redox stability, optimizable catalytic activity, as well as easy synthesis [9, 10, 11]. However, the single‐phase electrode has a simple chemical structure and limited O^2−^/e^−^ transport channels, which results in low catalytic activity [12], and thus low peak power density (PPD). To overcome such drawbacks, composite oxides containing two or more phases have been adopted as SOFCs anodes. For instance, Kong et al. successively immersed porous Sc_0.2_Zr_0.8_O_2‐δ_ anode into the precursor solutions of La_0.9_Ca_0.1_Fe_0.9_Nb_0.1_O_3‐δ_ (LCFNb), Sm_0.2_Ce_0.8_O_1.9_ (SDC), and Ni(NO_3_)_2_, and finally obtained the target anode LCFNb‐SDC‐Ni with rich multiphase interfaces and thus enhanced catalytic activity [13]. However, the growth of the exsolved particles at high temperature and prolonged operating time declined performance [14]. Recently, Lv et al. reported that the in situ reduction triggers B‐site exsolution with the phase reconstruction from simple perovskite to R‐P oxides (A_n+1_B_n_O_3n+1_), leading to improved redox stability and adequate MIEC [15, 16, 17]. For instance, the CoFe alloy nanoparticles (NPs) evenly and firmly anchored on the surface of Gd_2_SrCo_0.8_Fe_1.2_O_7‐δ_ (R‐P‐GSCF) can effectively avoid the grain coarsening at high temperature [18]. Meanwhile, the R‐P layered perovskite accommodates a large number of interstitial oxygen atoms, causing high concentration oxygen vacancies (*V_O_*) and thereby enhanced ionic conductivity [19]. However, the insufficient effective catalytic site at the tri‐phase boundary (TPB) often impedes the hydrogen oxidation reaction (HOR), and consequently restricts the overall reaction kinetics.

**FIGURE 1:**
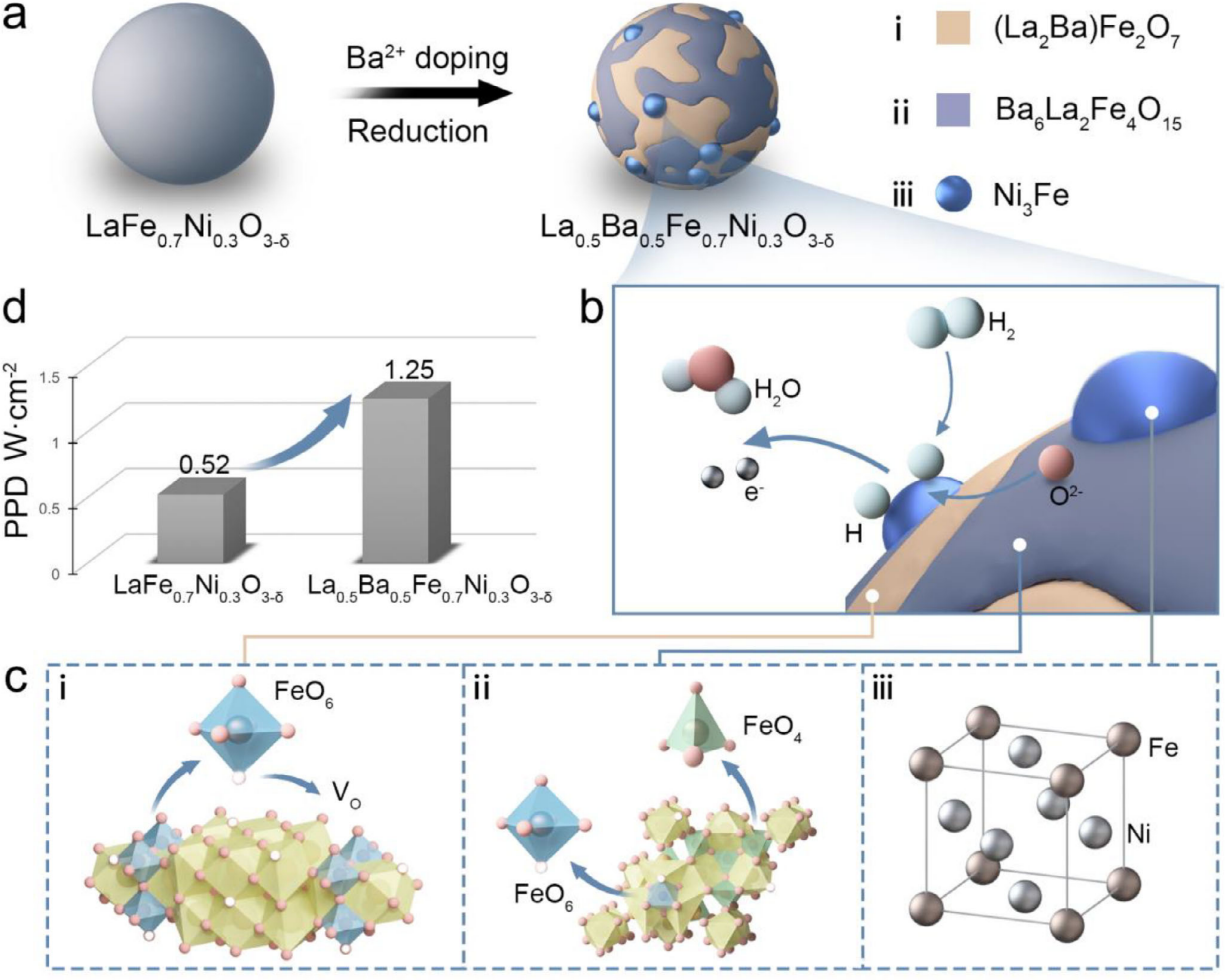
(a) Schematic diagram of phase transformation induced by Ba^2^^+^ doping and followed by reduction under H_2_ (3 vol% H_2_O) atmosphere. (b) Schematic of the electrochemical processes at the surface of LBFN‐0.5 anode. (c) Crystal structures of (La_2_Ba)Fe_2_O_7_, Ba_6_La_2_Fe_4_O_15_, and Ni_3_Fe NPs. (d) PPD comparison before and after Ba^2+^ doping at 800°C.

Herein, considering the excellent redox stability but relatively low ionic conductivity and deficient catalytic activity, we have developed a novel anode by purposively doping low electronegative Ba^2+^ at the La^3+^ site in LaFe_0.7_Ni_0.3_O_3‐δ_ (LFN), which is composed of (Figure 1a–c): (i) R‐P‐phase (La_2_Ba)Fe_2_O_7_ contributing to improved oxygen ion (O2‐) mobility due to the large number of *Vo* in the unique layered structure, (ii) hexagonal Ba_6_La_2_Fe_4_O_15_ offering additional FeO_4_ catalytic sites besides the intrinsic FeO_6_ ones in (La_2_Ba)Fe_2_O_7_ and facilitating rapid electron transfer, and (iii) the exsolved Ni_3_Fe NPs greatly accelerate HOR on the anode surface. Synergy of the above merits finally leads to a 57% polarization resistance (R_p_) reduction (from 0.28 to 0.12 Ω cm^2^), doubled PPD with a record value of 1.25 W cm^−2^ among Fe‐based analogous at 800°C (Figure 1d), a negligible voltage decrease rate of ∼8.9 × 10^−5^·V h^−1^, and a 300 h durability at 750°C.

## 2 Results and Discussion

The effect of different Ba^2+^ doping concentration at the La^3+^‐site on the crystal structure was investigated by X‐ray diffraction (XRD), where La_1‐x_Ba_x_Fe_0.7_Ni_0.3_O_3‐δ_ (LBFN‐x) sintered in air corresponds to a cubic perovskite structure at x = 0 mol (LBFN‐0) and x = 0.1 mol (LBFN‐0.1), then the R‐P‐(La_2_Ba)Fe_2_O_7_ phase emerges at x = 0.3 mol (LBFN‐0.3), and surprisingly, the third phase of hexagonal Ba_6_La_2_Fe_4_O_15_ appears at x = 0.5 mol (LBFN‐0.5) (Figure S1). Accordingly, La_2_O_3_ peak appears in LBFN‐0 after reduction under H_2_ (3 vol% H_2_O) atmosphere (Figures S2, S3 and Table S1), which impedes ion diffusion and electron conduction [20, 21, 22]. The disappearance of La_2_O_3_ in LBFN‐0 is attributed to the formation of the R‐P‐(La_2_Ba)Fe_2_O_7_ in LBFN‐x (x = 0.1‐0.3). Upon the introduction of Ba^2+^, it reacts with the excessive La_2_O_3_ and LaFeO_3_ presented in LBFN‐0 to form a thermodynamically more stable layered perovskite structure, and the single cubic perovskite transforms completely into R‐P‐(La_2_Ba)Fe_2_O_7_ in LBFN‐0.3. Due to the larger ionic radius of Ba^2+^ (1.61 Å) than La^3+^ (1.36 Å), the c‐axis of (La_2_Ba)Fe_2_O_7_ increases from 20.77 Å in LBFN‐0.1 to 20.81 Å in LBFN‐0.5. Meanwhile, a hexagonal Ba_6_La_2_Fe_4_O_15_ structure forms in LBFN‐0.5, which effectively accommodates the lattice distortion by providing a more flexible coordination environment. Furthermore, the Ni_3_Fe phase increases significantly to 16.00%. R‐P‐(La_2_Ba)Fe_2_O_7_ (PDF#72‐1743), Ba_6_La_2_Fe_4_O_15_ (PDF#86‐2298), as well as Ni_3_Fe (PDF#88‐1715) NPs are seen in LBFN‐0.5 after reduction under H_2_ (3 vol% H_2_O) at 800°C for 3 h (Figure S4). Continued increase of Ba^2+^ doping to x = 0.6 mol (LBFN‐0.6) leads to a sharp decrease in both the number of Ni_3_Fe NPs (Figure S5b) and conductivity than LBFN‐0.5 (Figure S6).

**FIGURE 2:**
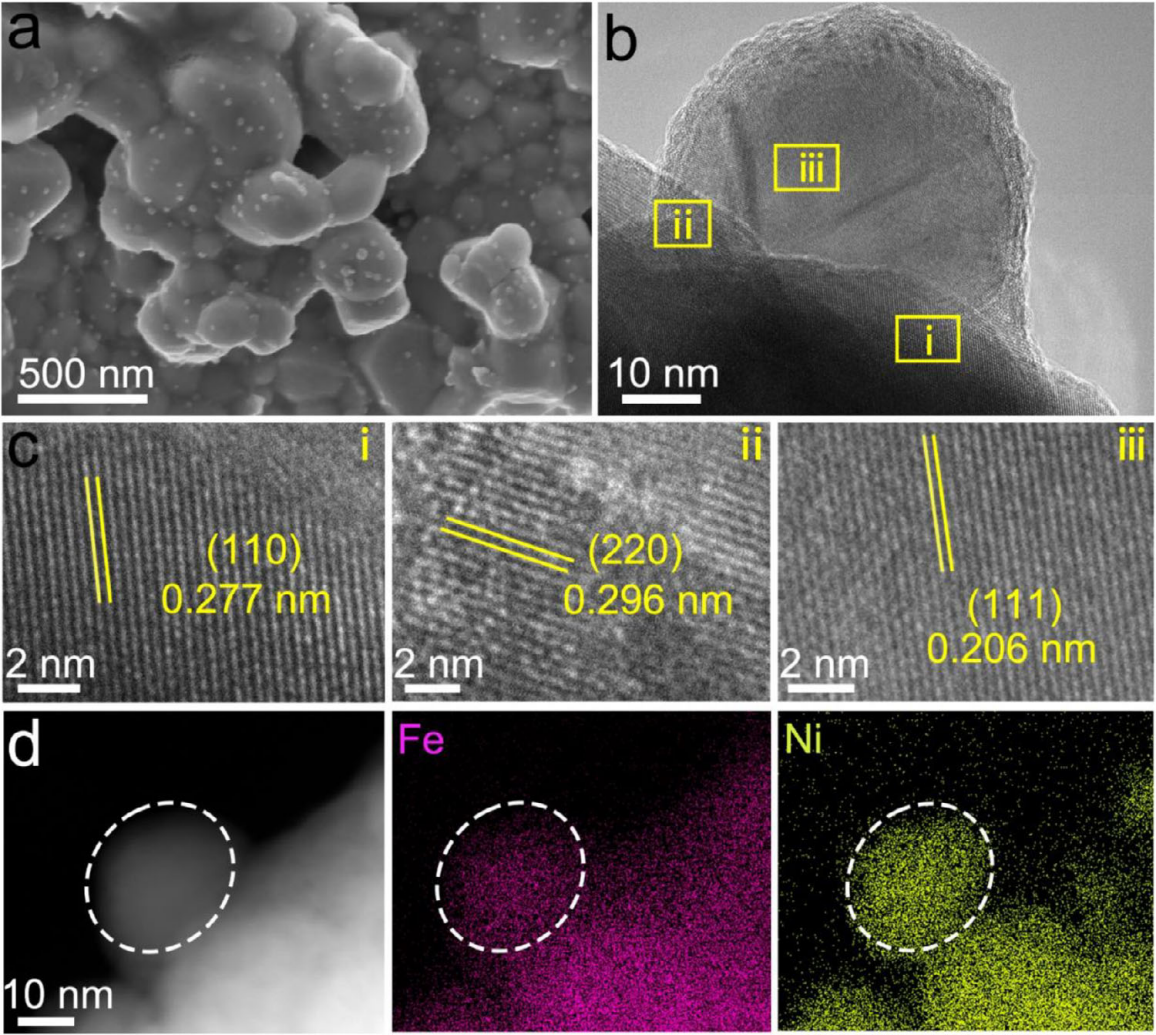
(a) SEM image of LBFN‐0.5 after reduction. (b) A typical exsolved Ni_3_Fe NPs on LBFN‐0.5 surface after reduction. (c) HRTEM image of reduced LBFN‐0.5 showing typical crystal lattice of (i) (La_2_Ba)Fe_2_O_7_, (ii) Ba_6_La_2_Fe_4_O_15_, and (iii) Ni_3_Fe NPs, respectively. (d) Elemental mapping of LBFN‐0.5 after reduction showing even distribution of Fe and Ni.

The scanning electron microscope (SEM) image shows ∼30 nm NPs on the surface of LBFN‐0.5 after reduction at 800°C in H_2_ (3 vol% H_2_O) (Figure 2a). The crystal planes of (110), (220), and (111) corresponding to (i) R‐P phase (La_2_Ba)Fe_2_O_7_, (ii) hexagonal phase Ba_6_La_2_Fe_4_O_15_, and (iii) cubic phase Ni_3_Fe NPs, respectively, are seen in the transmission electron microscope (TEM) images (Figure 2b,c), which form abundant tri‐phase intersection areas as illustrated in Figure 1c. The clear Fe and Ni signals in the energy dispersive X‐ray spectroscopy (EDX) mapping result suggest the formation of Ni_3_Fe NPs (Figure 2d; Figure S7). Consistently, the X‐ray photoelectron spectroscopy (XPS) signals of two deconvoluted subpeaks (Figure S8b), corresponding to Fe^2+^ (710.3 and 723.1 eV) and Fe^3+^ (713.4 and 726.2 eV), together with a new peak of Fe^0^ at 705.7 eV seen after reduction, imply successful exsolution of Fe (Figure S9) [23]. Similarly, the Ni^0^ peak at 66.5 eV after reduction (Figure S10b) proves the exsolved Ni_3_Fe NPs.

**FIGURE 3:**
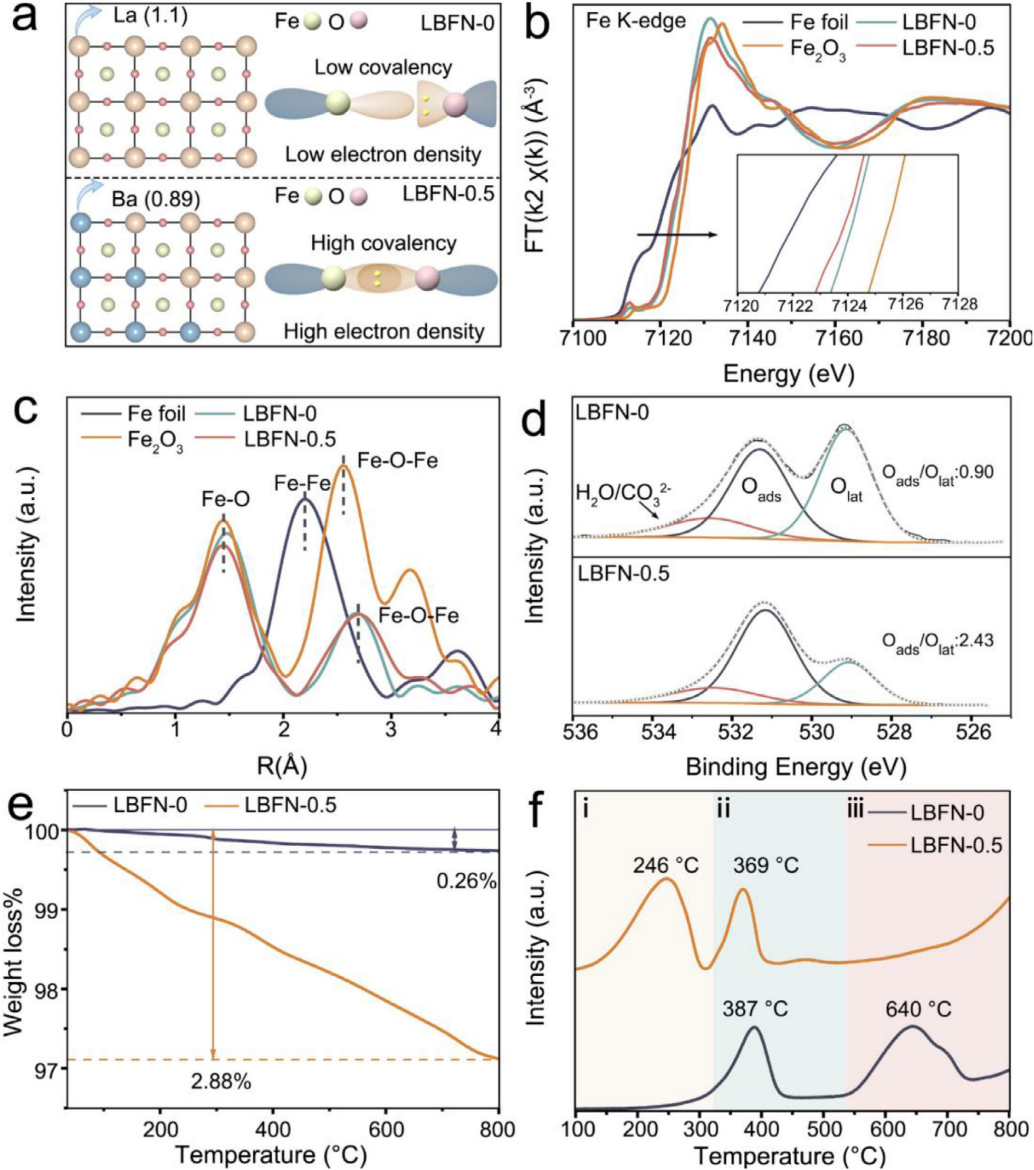
(a) Schematic illustration of the Fe─O bond covalency in LBFN‐0 and LBFN‐0.5. (b) Fe K‐edge XANES spectra of LBFN‐0 and LBFN‐0.5 before reduction. (c) Fourier transform EXAFS spectra of Fe K‐edge in LBFN‐0, LBFN‐0.5, and reference samples of Fe foil and Fe_2_O_3_, before reduction. (d) XPS spectra O 1s in LBFN‐0 and LBFN‐0.5 after reduction. (e) TGA spectra of LBFN‐0 and LBFN‐0.5 after reduction. (f) H_2_‐TPR results of LBFN‐0 and LBFN‐0.5.

*V_O_* formation or valence state increase at the B site usually occurs in order to compensate charge imbalance caused by doping of lower‐valent cations such as Sr^2+^ or Ba^2+^ at the A site [24, 25]. Moreover, the lower electronegativity of Ba^2+^ (0.89) compared with that of La^3+^ (1.1) can significantly reduce the electron attraction from neighboring O atoms from the connecting Fe atoms, leading to increased electron density on Fe atoms, as reflected by the lower average cation valence of Fe ion (Figure 3a; Table S2) [19, 25, 26]. Compared with the normalized X‐ray absorption near edge structures (XANES) spectra of LBFN‐0, shift of the absorption edge of Fe in LBFN‐0.5 towards low energy (Figure 3b) indicates a decrease of the oxidation state of Fe, which is consistent with the XPS analysis before (2.29 in LBFN‐0) and after (2.24 in LBFN‐0.5) Ba^2+^ doping (Table S2). The coordination sphere at 1.5 Å in the Fourier transform extended X‐ray absorption fine structure (EXAFS) spectrum of Fe K‐edge (Figure 3c) indicates the Fe─O scattering path, while that at 2.7 Å confirms the Fe─O─Fe scattering path [27]. According to the wavelet transform EXAFS (WT‐EXAFS) analysis, the coordination number of Fe decreases from 6.02 to 5.09 after Ba^2+^ doping, and the bond length reduces from 1.97 to 1.94 Å due to the significant increase in *V_O_* content, which results in distortion of the FeO_6_ octahedron, and finally formation of Ba_6_La_2_Fe_4_O_15_ containing more FeO_4_ catalytic sites at increased Ba^2+^ doping (Figure S11 and Table S3).

Deconvoluted O 1s XPS peaks at 529.0, 531.1, and 532.5 eV indicate the existence of lattice oxygen (O_lat_), adsorbed oxygen (O_ads_), and adsorbed H_2_O/CO_2_ (H_2_O/CO_3_^2−^_ads_), respectively (Figure 3d) [28, 29]. The oxygen activation capability of catalysts, characterized by the ratio of O_ads_ to O_lat_, increases from 0.90 in LBFN‐0 to 2.43 in LBFN‐0.5, suggesting increased *V_O_* along with the phase transition. The O 1s XPS analyses of (La_2_Ba)Fe_2_O_7_ and Ba_6_La_2_Fe_4_O_15_ show a higher O_ads_/O_lat_ ratio in (La_2_Ba)Fe_2_O_7_ (1.84) than that in Ba_6_La_2_Fe_4_O_15_ (1.54), suggesting that (La_2_Ba)Fe_2_O_7_ predominantly governs O^2−^ conduction in the tri‐phase structure (Figure S12). Consistently, thermogravimetric analyses (TGA) curves in N_2_ atmosphere (Figure 3e) show the mass loss at 800°C increases from 0.26% in LBFN‐0 to 2.88% in LBFN‐0.5, implying increased release of O_lat_, i.e., formation of more *V_O_* after Ba^2+^ doping [30].

H_2_‐temperature‐programmed reduction (H_2_‐TPR) profiles can reveal the metallic nature of Ni and Fe, as well as their interactions with the bulk material (Figure 3f). Each H_2_ consumption peak corresponds to an independent reduction process. The peak appears in region i corresponds to the transition from Fe^3+^ to Fe^2+^ for LBFN‐0.5, the peak in region ii corresponds to the reduction of Ni^2+^ to Ni^0^ and Fe^2+^ to Fe^0^ for LBFN‐0.5 and Ni^2+^ to Ni^0^ and Fe^3+^ to Fe^2+^ for LBFN‐0, while the peak in region iii corresponds reduction of Fe^2+^ to Fe^0^ for LBFN‐0 [31]. The hydrogen consumption peak gradually shifts to a lower temperature with the phase structure transition, indicating that the interaction between Ni^2+^, Fe^3+^, and the bulk material is weakened and thus the reduction process becomes easier after Ba^2+^ doping [32, 33].

The 55% R_p_ reduction from 0.29 Ω cm^2^ in LBFN‐0 to 0.13 Ω cm^2^ in LBFN‐0.5 measured in the symmetrical cell (Figures S13 and S14) at 800°C under H_2_ (3 vol% H_2_O) atmosphere, highlights the synergistic effect of the *V_O_*, FeO_6_/FeO_4_ coordination environments, and Ni_3_Fe NPs in the tri‐phase structure, which significantly enhances the HOR catalytic activity. Decrease of the activation energy of the anode from 1.03 in LBFN‐0 to 0.78 eV in LBFN‐0.5 (Figure S15) indicates improved reaction kinetics after Ba^2+^ doping.

**FIGURE 4:**
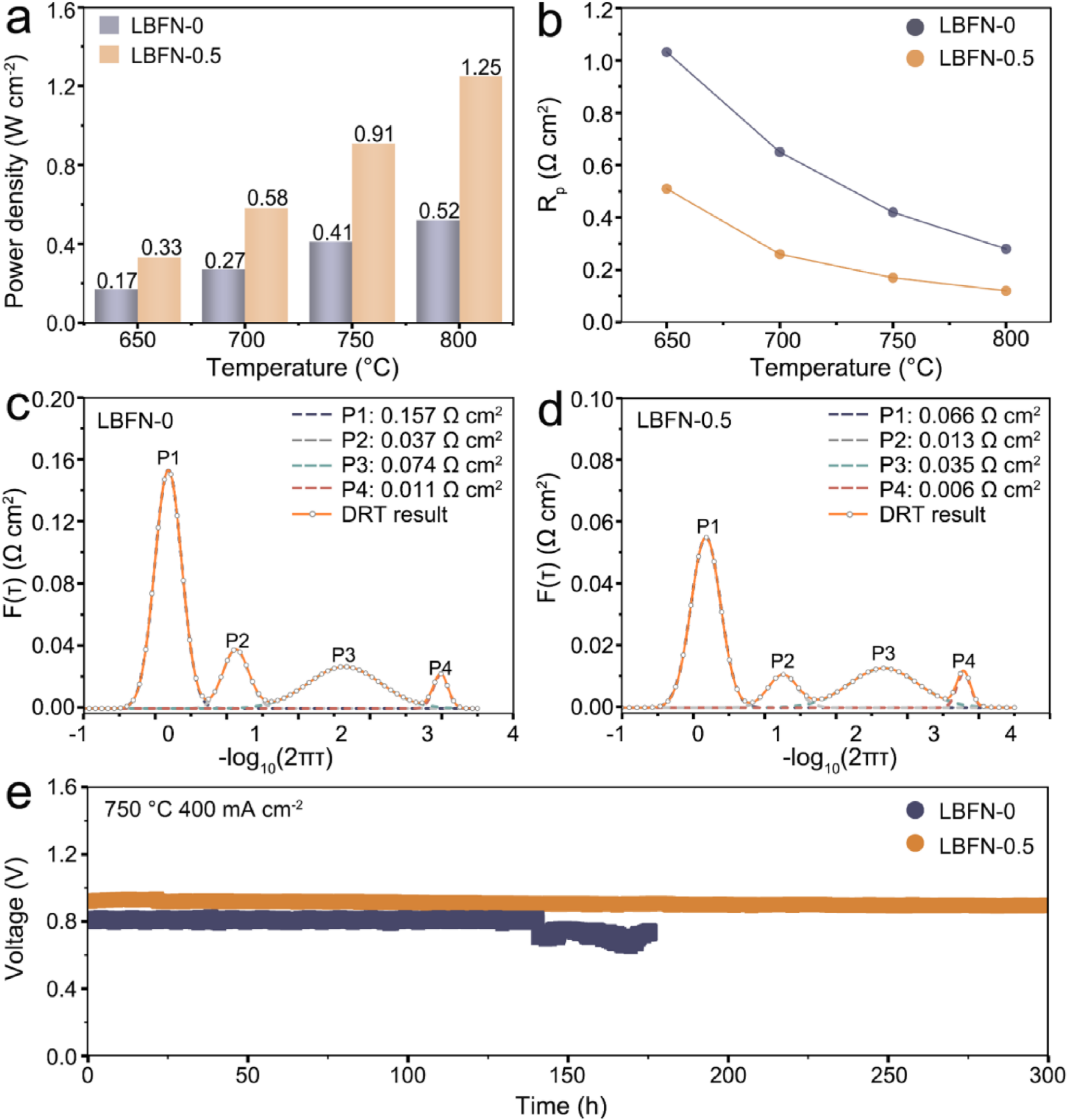
(a) PPD and (b) R_p_ comparison of LBFN‐0 and LBFN‐0.5 at 800°C–650°C of a single cell. DRT analysis of (c) LBFN‐0 and (d) LBFN‐0.5 at 800°C in H_2_ (3 vol% H_2_O) of a single cell. (e) Stability test of the single cell using LBFN‐0 and LBFN‐0.5 as anode at 750°C and 400 mA cm^−2^.

The 1.25 W cm^−2^ PPD of the single cell at 800°C with a synergized anode of LBFN‐0.5, i.e., R‐P‐(La_2_Ba)Fe_2_O_7_/Ba_6_La_2_Fe_4_O_15_/Ni_3_Fe, shows a 140% improvement compared with that of 0.52 W cm^−2^ in LBFN‐0 (Figure 4a; Figure S16), representing so far the highest value among Fe‐based analogous (Table S4). Decrease of the R_p_ from 0.28 to 0.12 Ω cm^2^ when the Ba^2+^ doping concentration increases from 0 to 0.5 mol (Figure 4b) means improved catalytic activity of the resulting anode (Figure S17).

The four distributions of relaxation times (DRT) peaks (Figure 4c,d) in the low (P1), intermediate (P2, P3), and high frequency ranges (P4), correspond to gas diffusion, ion surface exchange, and interface charge transfer processes, respectively [33, 34]. The low and intermediate frequencies are likely related to hydrogen adsorption/desorption, dissociation, and surface transport. The polarization impedance decreases from 0.16 to 0.07 Ω cm^2^ and 0.11 to 0.05 Ω cm^2^ at low and intermediate frequency, respectively, as the dual‐phase LBFN‐0 transforms to tri‐phase LBFN‐0.5. The trivial impedance change (from 0.011 in LBFN‐0 to 0.006 Ω cm^2^ in LBFN‐0.5) in the high‐frequency range indicates that charge transfer is not the rate‐limiting step in the anode reaction [35, 36, 37]. The higher *V_O_* concentration in R‐P‐(La_2_Ba)Fe_2_O_7_/Ba_6_La_2_Fe_4_O_15_ enable better O^2−^ transport to the reaction area, thereby accelerating the hydrogen surface exchange process (Figure S18). In addition, this reduction in Fe ion size (from 0.645 in FeO_6_ to 0.49 Å in FeO_4_) caused by the low coordination environment directly shortens the Fe─O bond length from 2.045 to 1.89 Å.

According to the bonding principle, this more compact bonding geometry significantly enhances the spatial overlap between the Fe 3d and O 2p orbitals, thereby imparts the tetrahedral FeO_4_ with more covalency than the octahedral FeO_6_ [38, 39]. This well consists with the shorter average bond lengths in the EXAFS results (Figure S19), effectively promotes hydrogen adsorption and dissociation, and highly favorites catalytic oxidation reactions [40, 41, 42]. Parallelly, the unfilled *d* orbitals in Ni_3_Fe prompts Fe─H/Ni─H coordination bond formation with H_2_, which reduces the activation energy of the reaction and enhances hydrogen adsorption [43]. In order to clarify the contribution of each phase, combined with the DRT analysis of the symmetrical cells and work function result, the lowest work function of (La_2_Ba)Fe_2_O_7_ (4.66 eV) among the three individual phases indicates the lowest *V_O_* formation energy, primarily contributing to the promotion of O^2−^ conduction. The work function of Ba_6_La_2_Fe_4_O_15_ (4.68 eV) matches well with that of (La_2_Ba)Fe_2_O_7_ and Ni_3_Fe, which improves electron transfer. Furthermore, the highest work function of Ni_3_Fe (4.69 eV) among the three individual phases helps increase electron density and reduce the energy barrier for hydrogen adsorption and dissociation. The synergistic effect of the tri‐phase endows reduced LBFN‐0.5 (4.65 eV) with the lowest work function (Figures S20–S23) [44, 45]. As a result, the designed tri‐phase structure makes full use of the synergistic effect to catalyze H_2_ oxidation. The negligible voltage degradation when running at a constant current of 400 mA cm^−2^ at 750°C for 300 h suggests excellent catalytic stability (Figure 4e).

## 3 Conclusions

A high‐performance, tri‐phase anode material composed of R‐P‐(La_2_Ba)Fe_2_O_7_, hexagonal Ba_6_La_2_Fe_4_O_15_, and cubic Ni_3_Fe NPs for SOFC has been developed by purposive Ba^2+^ doping into the La^3+^ site of the conventional LaFe_0.7_Ni_0.3_O_3‐δ_. The as‐fabricated single cell shows a record‐high PPD of 1.25 W cm^−2^ among Fe‐based analogues, which can be attributed to the synergistic effect of rapid supply of O^2−^ by R‐P‐(La_2_Ba)Fe_2_O_7_, and the additional catalytic sites provided by hexagonal Ba_6_La_2_Fe_4_O_15_, as well as improved adsorption and activation of H_2_ by Ni_3_Fe NPs. This study paves a novel path to developing stable and efficient catalysts as anodes for SOFC.

## 4 Experimental Section

Experimental details are included in the Supporting Information.

## Conflicts of Interest

The authors declare no conflicts of interest.

## Supporting information




**Supporting File**: advs75578‐sup‐0001‐SuppMat.docx.

## Data Availability

The data that supports the findings of this study are available in the supplementary material of this article.
