# Supplementary material for: Synergized Tri‐Phase Anode via Purposive Ba2+ Doping at the La3+ Site in LaFe0.7Ni0.3O3‐δ for Improved SOFC Performance
Source: Adv Sci (Weinh). 2026 May 8;13(43):e75578. doi: 10.1002/advs.75578 (PMC13335915; doi:10.1002/advs.75578)
Supplement: Supplementary file 1 — Supporting File: advs75578‐sup‐0001‐SuppMat.docx. [file ADVS-13-e75578-s001.docx]

Supporting Information

**Synergized Tri-phase Anode via Purposive Ba^2+^ Doping at the La^3+^ Site in LaFe_0.7_Ni_0.3_O_3-δ_ for Improved SOFC Performance**

Qian Yang^1^, Shuaihang Chen^1^, Yongli Peng^1^, Xuelong Li^1^, Xiaojian Pan^1^, Fang Wang^1^, Nanya Long^1^, Li Li^1^, Yilin Wang^2^, Lina Su^1,^*, Yuesong Shen^2,^*, Ling Huang^1,2,^*

^1^State Key Laboratory of Chemistry and Utilization of Carbon Based Energy Resources, College of Chemistry, Xinjiang University, Urumqi 830046, China.

^2^School of Materials Science and Engineering, Nanjing Tech University, Nanjing 211816, China.

E-mail: [sulina@xju.edu.cn](mailto:sulina@xju.edu.cn); [shenyuesong@njtech.edu.cn](mailto:shenyuesong@njtech.edu.cn); [iamlhuang@njtech.edu.cn](mailto:iamlhuang@njtech.edu.cn)

**Experimental Section/Methods**

**Materials synthesis and cell fabrication.** La_1-x_Ba_x_Fe_0.7_Ni_0.3_O_3_ samples (x = 0, 0.1, 0.3, 0.5, 0.6) were synthesized by the Sol-Gel method. For LBFN-x materials, stoichiometric amounts of La(NO_3_)_3_·6H_2_O (Aldrich, 99.9%), Ba(NO_3_)_2_ (Aldrich, 99%), Fe(NO_3_)_3_·9H_2_O (Aldrich, 99.99%), Ni(NO_3_)_2_ (Aldrich, 99.9%), and Ethylenediaminetetraacetic acid (EDTA) were added to deionized water, mixture under continuous stirring, followed by the addition of citric acid, the molar ratio of total metal ions: EDTA: citric acid was 1:1:1.5, NH_3_·H_2_O was added dropwise to maintain a pH at ~8. The solution was slowly evaporated at 80 °C until a homogeneous organic/cation resins was achieved. After removal of excess resin by heating at 200 °C, black organic precursors containing metals were formed. The precursors were sintered at 1100 °C for 5 h in air environment. The synthesized sample was treated at 800 °C for 3 h under H_2_ (3 vol% H_2_O). La_0.8_Sr_0.2_Ga_0.8_Mg_0.2_O_3-δ_ (LSGM) powder was prepared by conventional solid-state reaction to fabricate LSGM-supported symmetrical cell and single cell. Stoichiometric amounts of La_2_O_3_ (Sigma, 99.99%), SrCO_3_ (Sigma, 99.99%), Ga_2_O_3_ (Sigma, 99.99%), and MgO (Sigma, 99.9%) powders were first mixed in a mortar and then ground in ethanol for 6 h to obtain the desired composition. After drying, the obtained powder was calcined at 1100 °C for 10 h. After formation of LSGM powder with desired stoichiometry, the electrolyte substrate was prepared by pressing at 2 MPa for 60 s into cylindrical shape and then sintered at 1450 °C for 6 h. The thickness of LSGM electrolyte was polished to about 250 μm. La_0.6_Sr_0.4_Co_0.2_Fe_0.8_O_3_ (LSCF) was used as the cathode, prepared using the glycine nitrate method. The stoichiometric ratio of La(NO_3_)_3_ (Sigma, 99.99%), Sr(NO_3_)_2_ (Sigma, 99.99%), Co(NO_3_)_2_ (Sigma, 99.99%), and Fe(NO_3_)_3_ (Sigma, 99.99%) was dissolved in the appropriate amount of water and then glycine was added, and the gel was formed by stirring and heating at 80 °C and then transferred to a universal furnace and heated to form a fluffy powder precursor, which was then calcined at 900 °C for 5 h to obtain the final product. La_0.4_Ce_0.6_O_2-δ_ (LDC) as a buffer layer was synthesized using the glycine nitrate method. The stoichiometric ratio of La(NO_3_)_3_ (Sigma, 99.99%), Ce(NO_3_)_3_ (Sigma, 99.99%) was put into a beaker, appropriate amount of water was added, and when the nitrate was dissolved, glycine was added, at the ratio of nitrate to glycine was 1:2. The gel was formed by stirring at 80 °C and then transferred to a universal furnace and heated until a fluffy powder precursor was formed, and the precursor was calcined at 700 °C for 3 h to obtain the final product.

**Cell Preparation**. To investigate the electrochemical performance of LBFN-x anode materials with different Ba^2+^ contents, we fabricated electrolyte-supported symmetric cells (LBFN-x|LDC|LSGM|LDC|LBFN-x) and single cells (LBFN-x|LDC|LSGM|LSCF). In the preparation of the symmetric cell, the LDC layer was screen-printed between the electrode and the electrolyte to prevent ionic species from diffusing into each other between the electrode and the electrolyte, and calcined at 1300 °C for 2 h. The anode powder with added binder was ground uniformly and coated on both sides of the LSGM electrolyte printed with the LDC buffer layer, and calcined at 1150 °C for 2 h, to obtain the symmetric cell. In the preparation of the single cell, the binder is added to the LBFN-x powder and ground into a uniform slurry, and is coated on the LDC buffer layer, and then calcined at 1150 °C for 2 h, the cathode slurry is synthesized in the same way, and then coated on the other side of the LSGM, and calcined at 1100 °C for 2 h. The effective area of the cathode side is 0.28 cm^2^, and the single cell LBFN-x|LDC|LSGM|LSCF is obtained.

**Structural characterization**. The initial crystal structures of the LBFN-x were characterized by XRD (Rigaku TTR-III diffractometer) with Cu Kα (λ = 1.5418 Å) radiation ranging at 20-70°. The microstructures of the anode powders and the fabricated single cells were observed with SEM (S-4800). The elemental surface analysis was performed using XPS (Thermo Scientific K-Alpha, USA). TEM (JEOL ARM-200F) was used to investigate the fine structure of the powders. TGA is used to assess how the mass of a material changes with temperature, thereby obtaining information such as thermal stability and changes in high temperature oxygen content of the material. In this paper, STA449F3 synchronous thermal analyzer of Netzsch STA 449 F5 was used for testing. The test temperature range was from room temperature to target temperature (as required), and the heating rate was 10 °C min^-1^. H_2_-TPR test is used to determine the change of hydrogen concentration with temperature during the reduction process of the material, so as to obtain the reduction temperature and content of each component in the material. In this paper, the American Micromeritics AutoChem II 2920 instrument was used for testing. The XAFS spectra of LBFN-0, LBFN-0.5, and Fe K-edge for two references (Fe foil and Fe_2_O_3_ powder) before reduction were measured in transmission mode at the Shanghai Synchrotron Radiation Facility (SSRF). XAFS and Fourier Transform (FT) EXAFS spectral analysis was performed using the Athena (Demeter) program. The surface potential of different phases was measured by Kelvin probe force microscopy (KPFM). The tip model used was SCM-PIT-V2. The bias voltage was applied to the tip. The standard value for **highly oriented pyrolytic graphite (**HOPG) is 4.60 eV. The work function calculation formula is:

$$V_{CPD}=\frac{\varphi_{s}-\varphi_{t}}{e}$$

among them, $V_{CPD}$ represents surface potential, $\varphi_{s}$is the work function of the sample, and $\varphi_{t}$ is the work function of the tip.

**Electrochemical performance measurements**. The electrochemical performances of the symmetrical cells were tested using an electrochemical workstation (GAMRY interface 5000). Electrochemical impedance spectra (EIS) measurements were made over a frequency range of 1 × 10^6^ to 0.1 Hz with a bias voltage of 20 mV. The electrochemical performances of the single cells were tested using an Energylab XM electrochemical workstation. EIS measurements were made over a frequency range of 1 × 10^6^ to 0.01 Hz with a bias voltage of 20 mV. The LBFN-x|LDC|LSGM|LSCF single cell was mounted on an alumina tube using a ceramic adhesive with the cathode side exposed to ambient air and the anode chamber with H_2_ (3 vol% H_2_O). Silver paste and silver wire were used for the connection of the fuel and air electrodes. The test temperature range of symmetric cells and single cells is 650-800 °C (interval temperature: 50 °C), and the gas flow rate is 100 ml min^-1^.


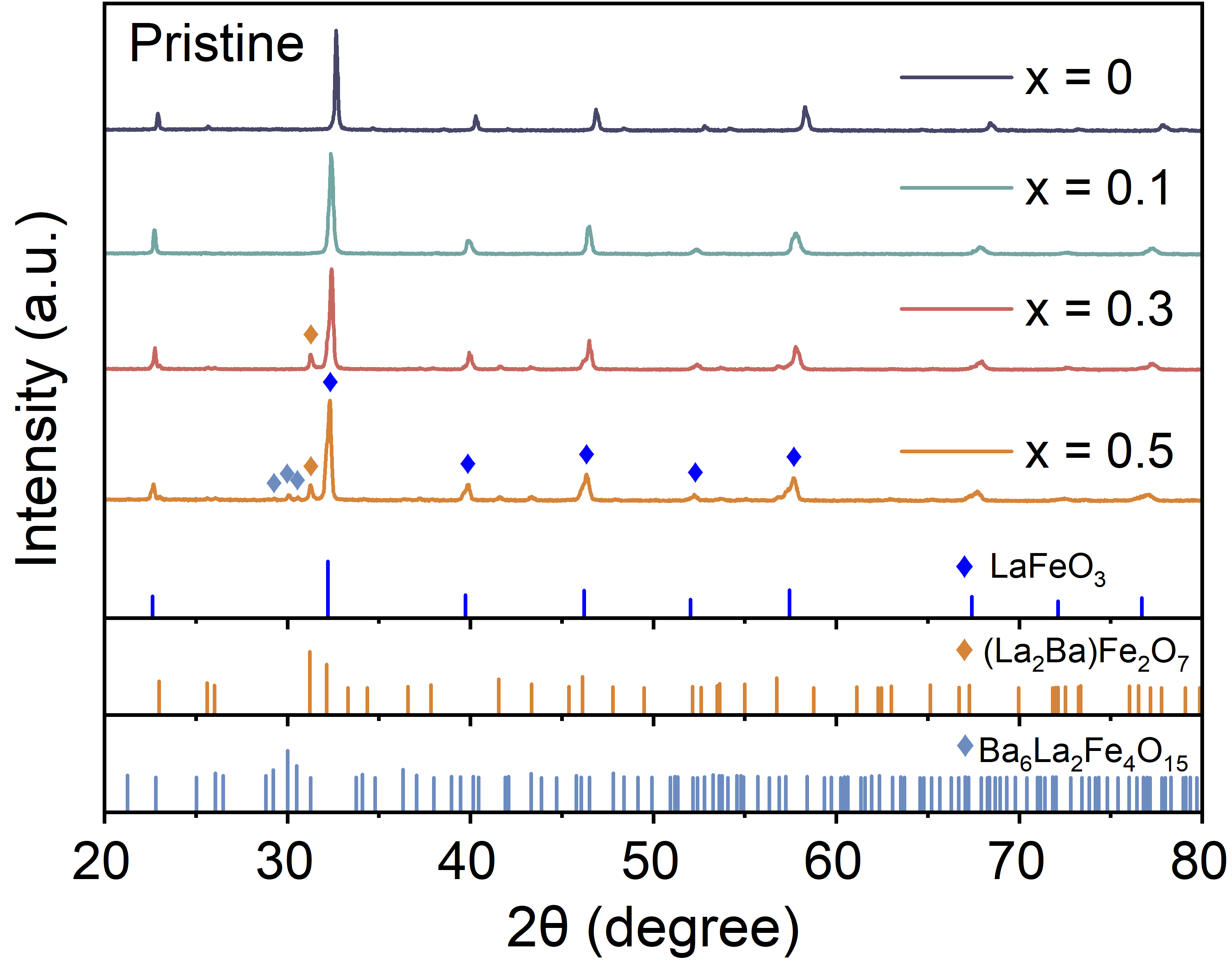


**Figure S1**. XRD pattern of the sample before reduction.

**
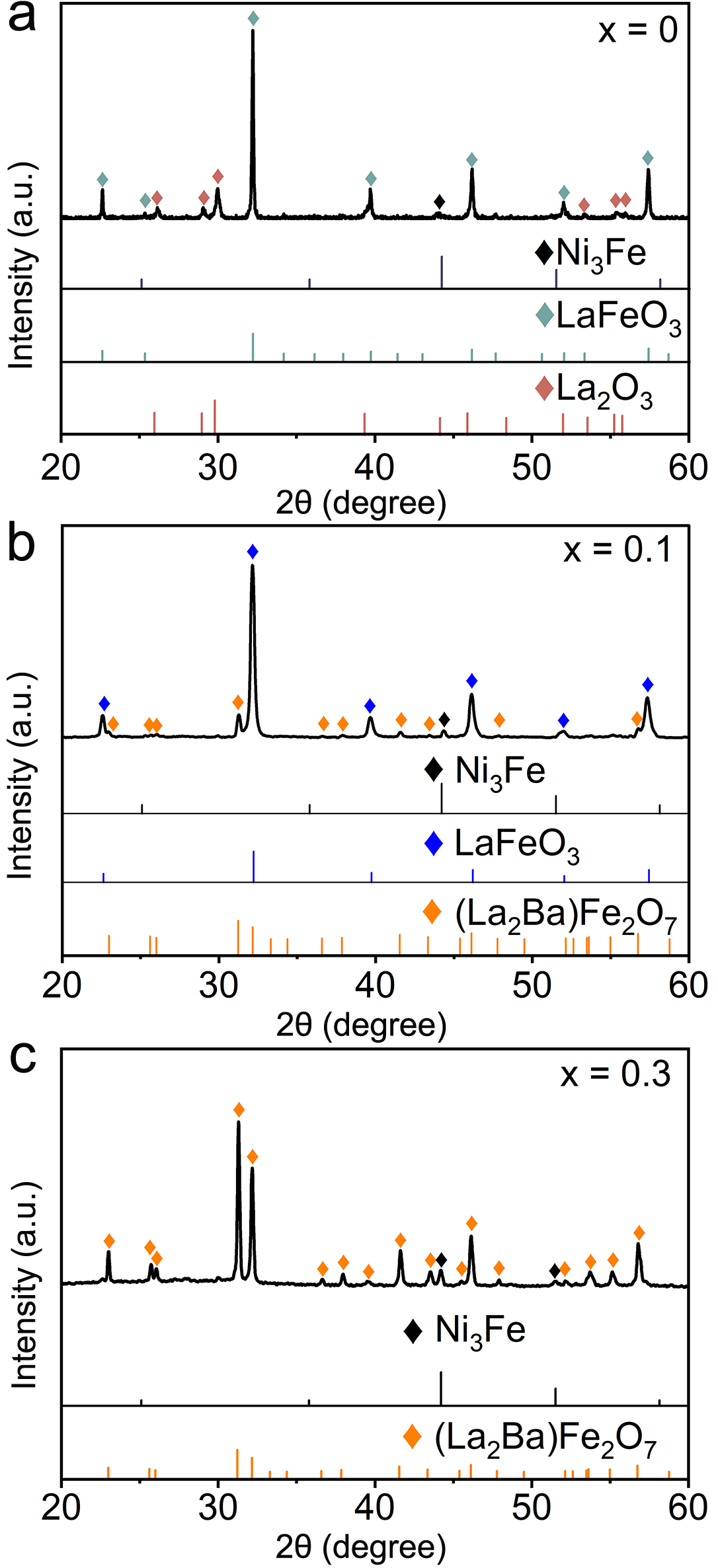
**

**Figure S2**. XRD patterns of anode La_1-x_Ba_x_Fe_0.7_Ni_0.3_O_3-δ_ (x = 0, 0.1, 0.3) after reduction under H_2_ (3 vol% H_2_O) at 800 °C for 3 h.

**
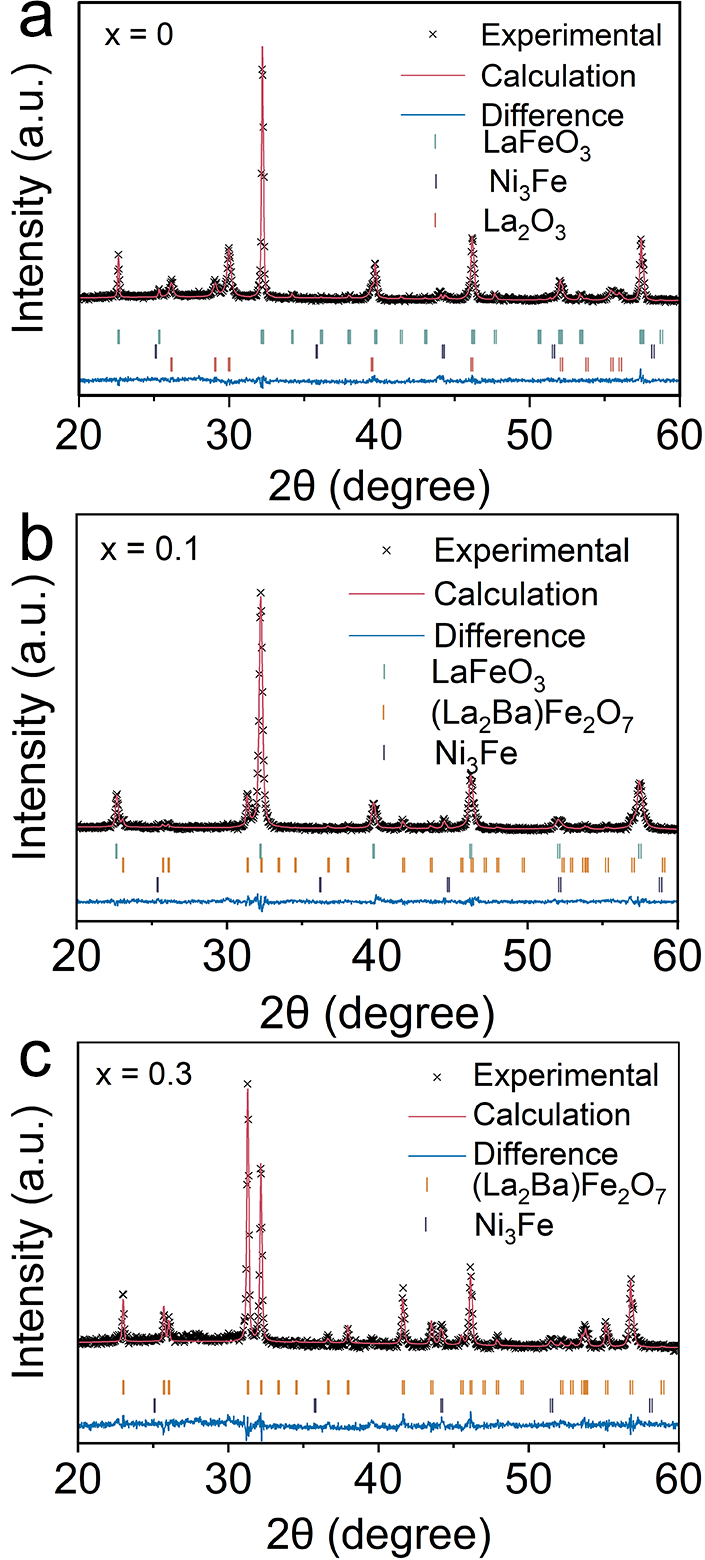
**

**Figure S3**. Refined XRD patterns of La_1-x_Ba_x_Fe_0.7_Ni_0.3_O_3-δ_ (x = 0, 0.1, 0.3) anode after reduction under H_2_ (3 vol% H_2_O) at 800 °C for 3 h.


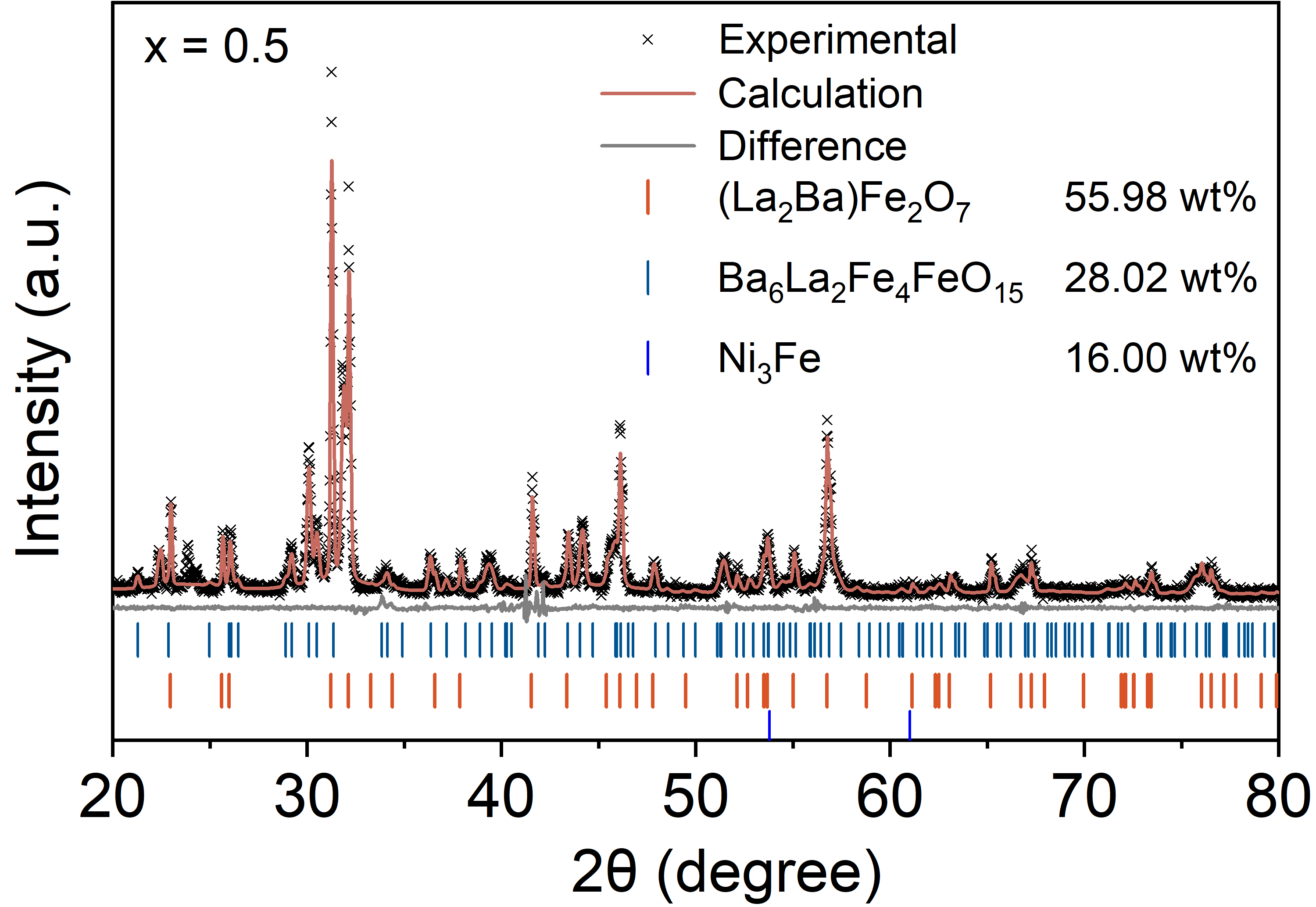


**Figure S4**. XRD refined patterns of anode LBFN-0.5 after reduction under H_2_ (3 vol% H_2_O) at 800 °C for 3 h.


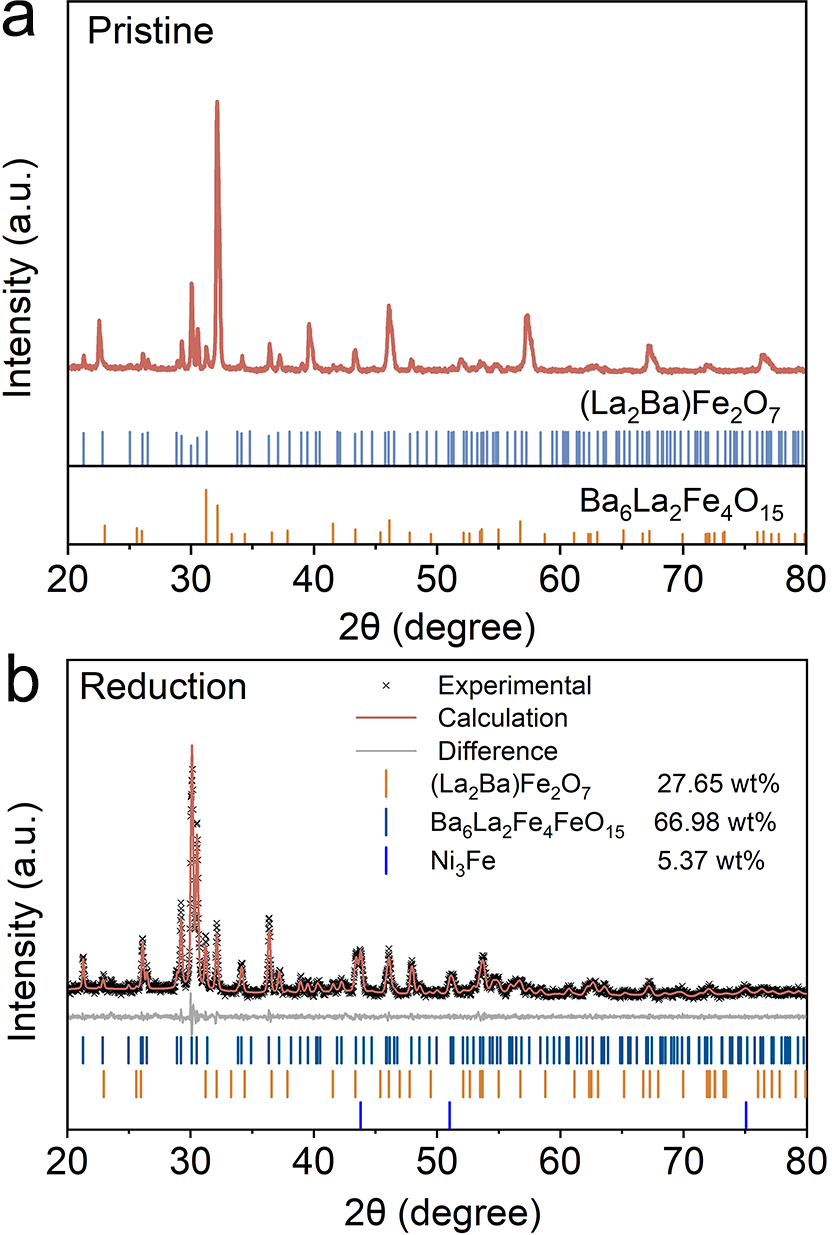


**Figure S5**. a) XRD and b) refined XRD patterns of the LBFN-0.6 anode before and after reduction under H_2_ (3 vol% H_2_O) at 800 °C for 3 h.


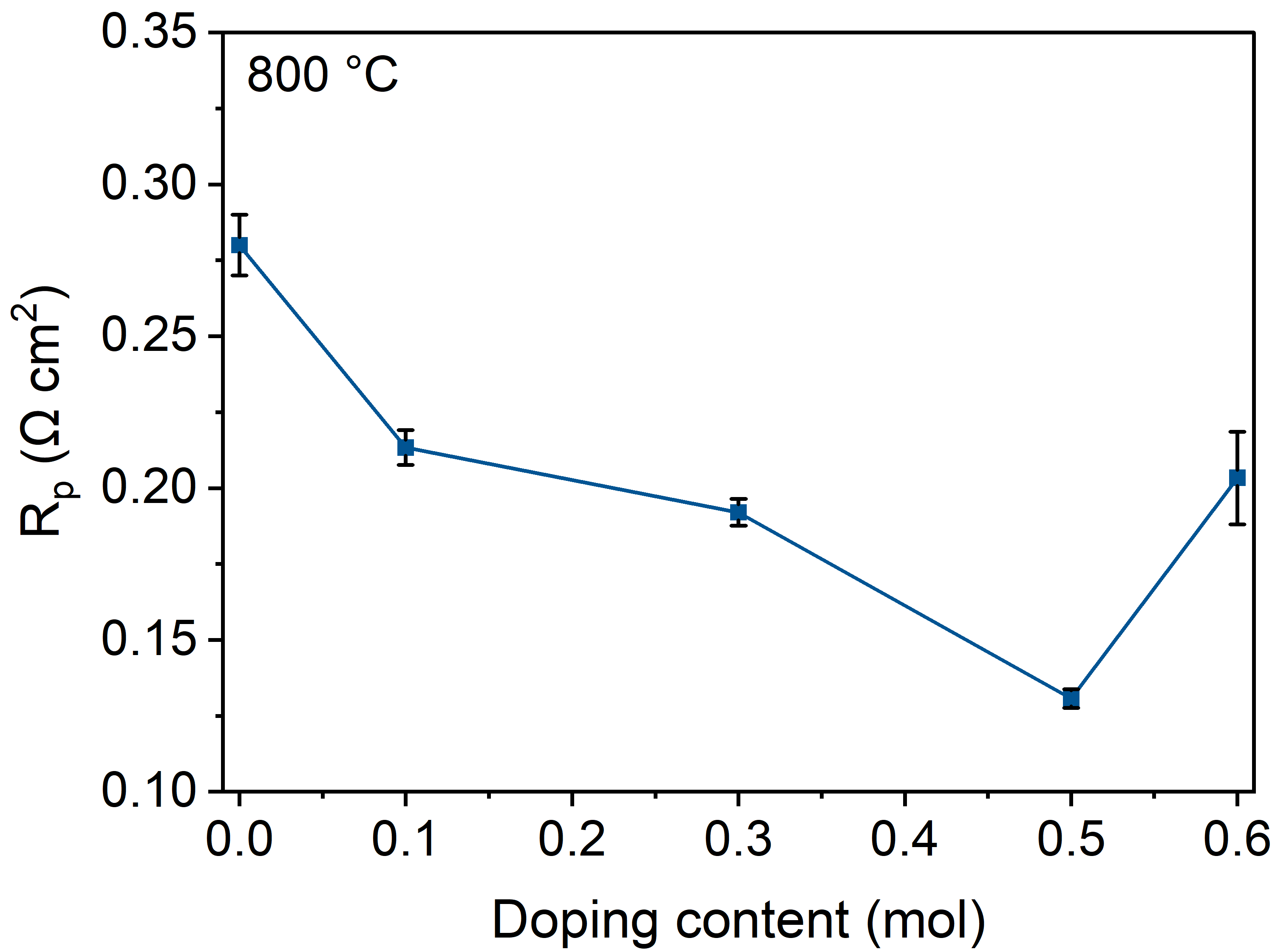


**Figure S6**. R_p_ analysis of La_1-x_Ba_x_Fe_0.7_Ni_0.3_O_3-δ_ (x = 0, 0.1, 0.3, 0.5, 0.6) at 800 ℃ under H_2_ (3 vol% H_2_O).


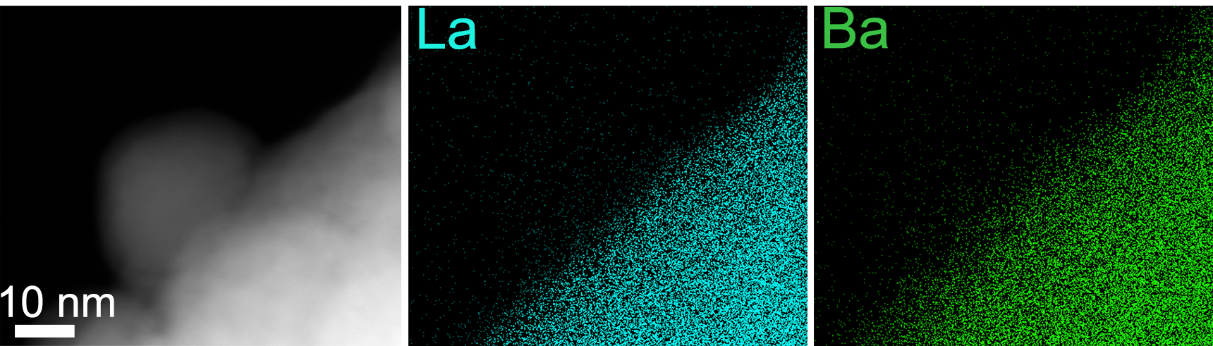


**Figure S7**. Elemental mapping of LBFN-0.5 after reduction.


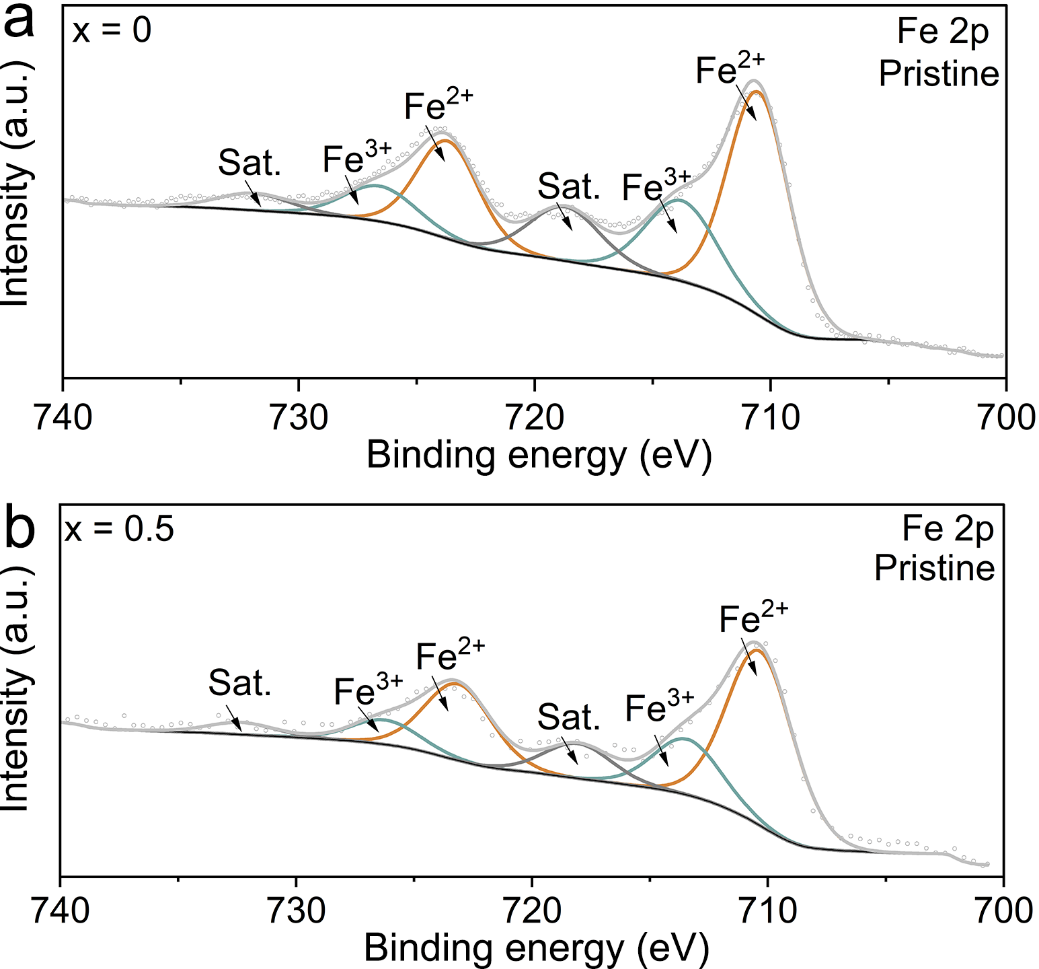


**Figure S8**. XPS image of Fe 2p for a) LBFN-0 and b) LBFN-0.5 before reduction.


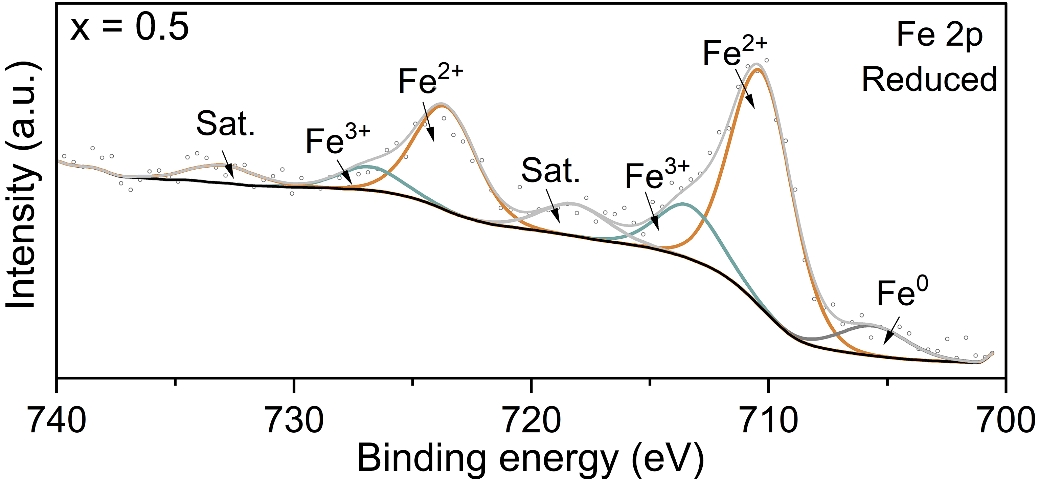


**Figure S9**. XPS profile of LBFN-0.5 after reduction.


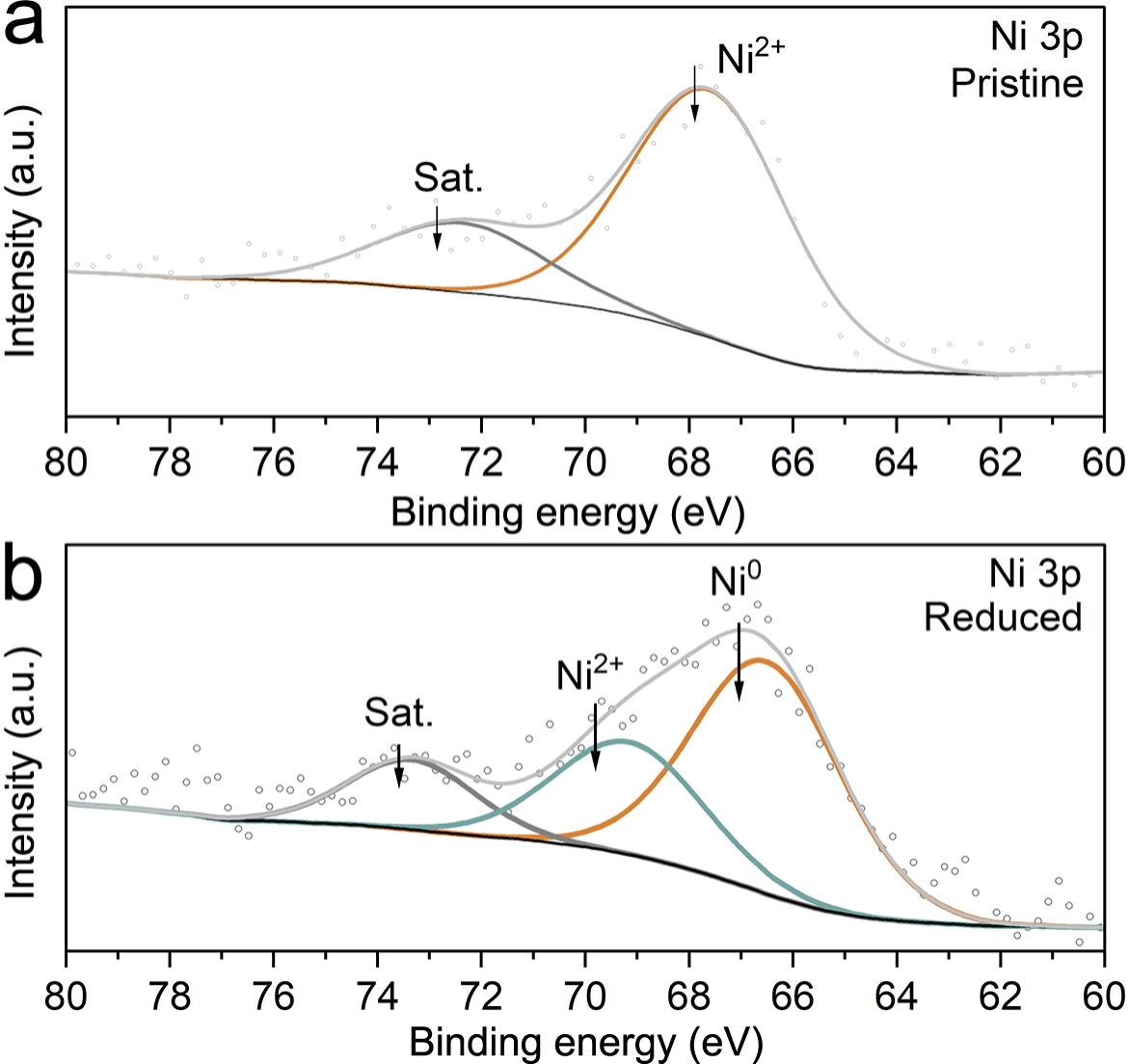


**Figure S10**. XPS image of Ni 3p for LBFN-0.5 a) before reduction, b) after reduction.


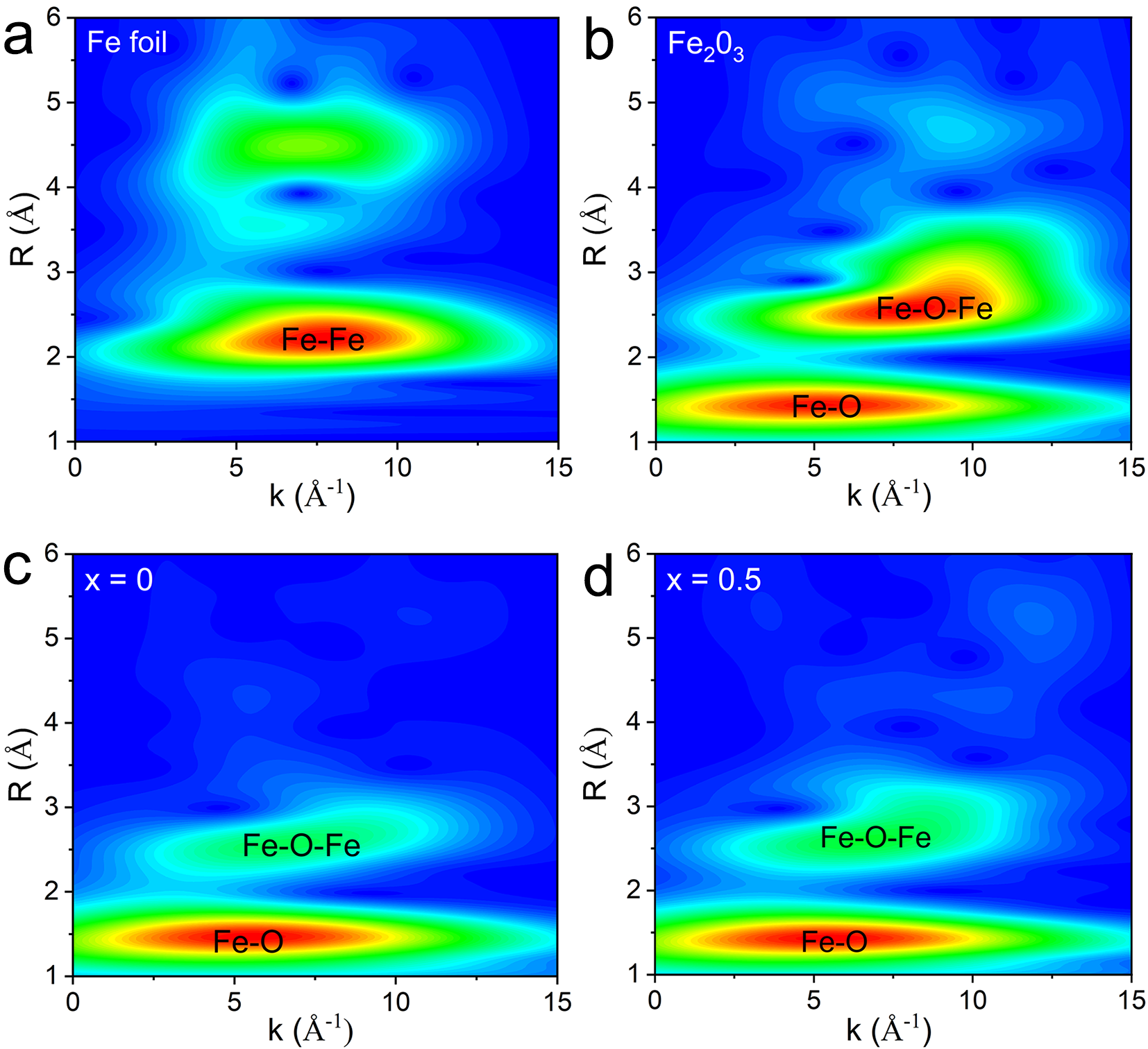


**Figure S11**. Wavelet transforms of the k3-weighted Fe K-edge EXAFS spectra of Fe foil, Fe_2_O_3_, LBFN-0, and LBFN-0.5.


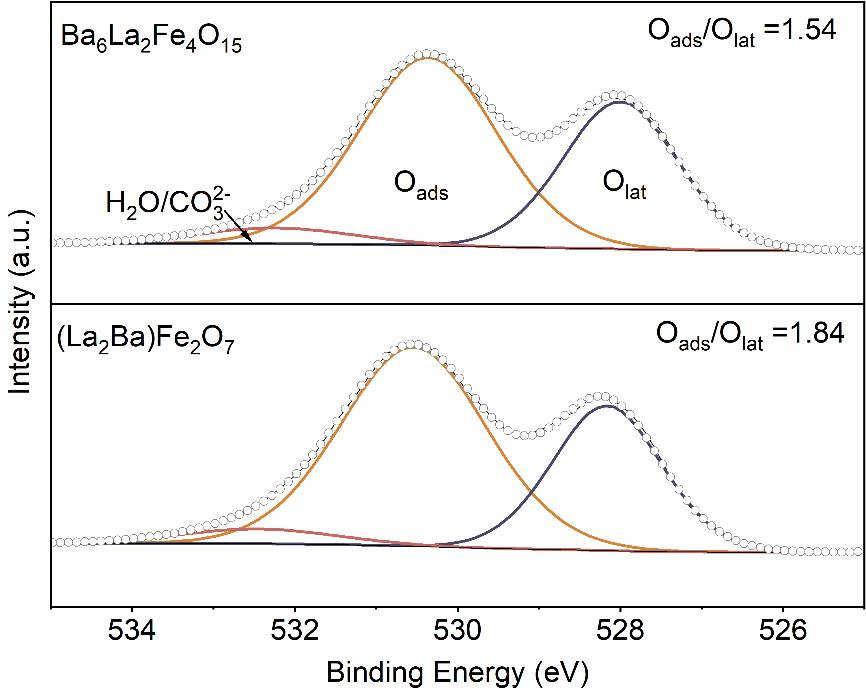


**Figure S12**. O 1s XPS spectra of (La_2_Ba)Fe_2_O_7_ and Ba_6_La_2_Fe_4_O_15_.


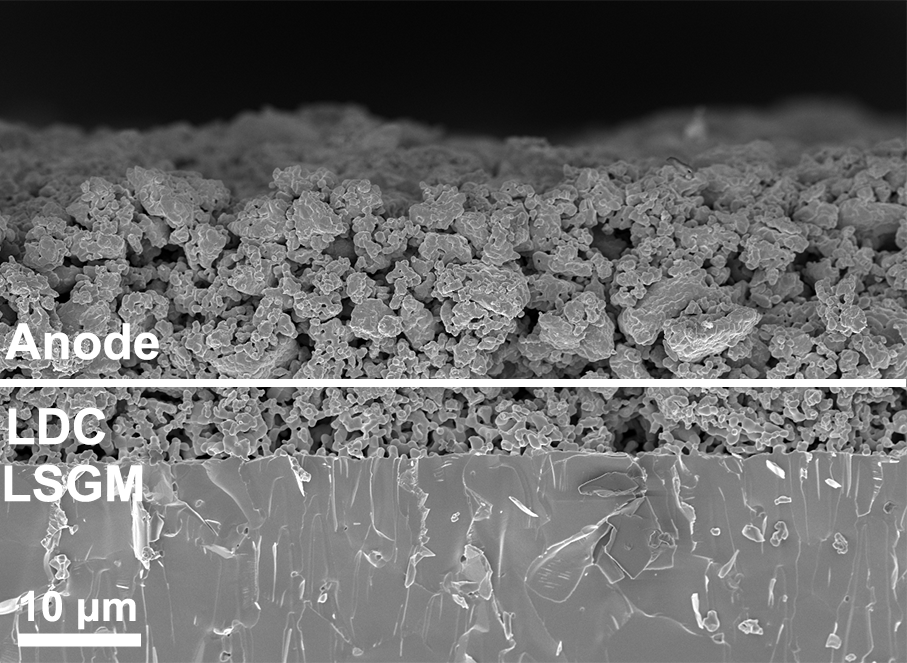


**Figure S13**. LSGM-supported symmetrical cell structure image.

**
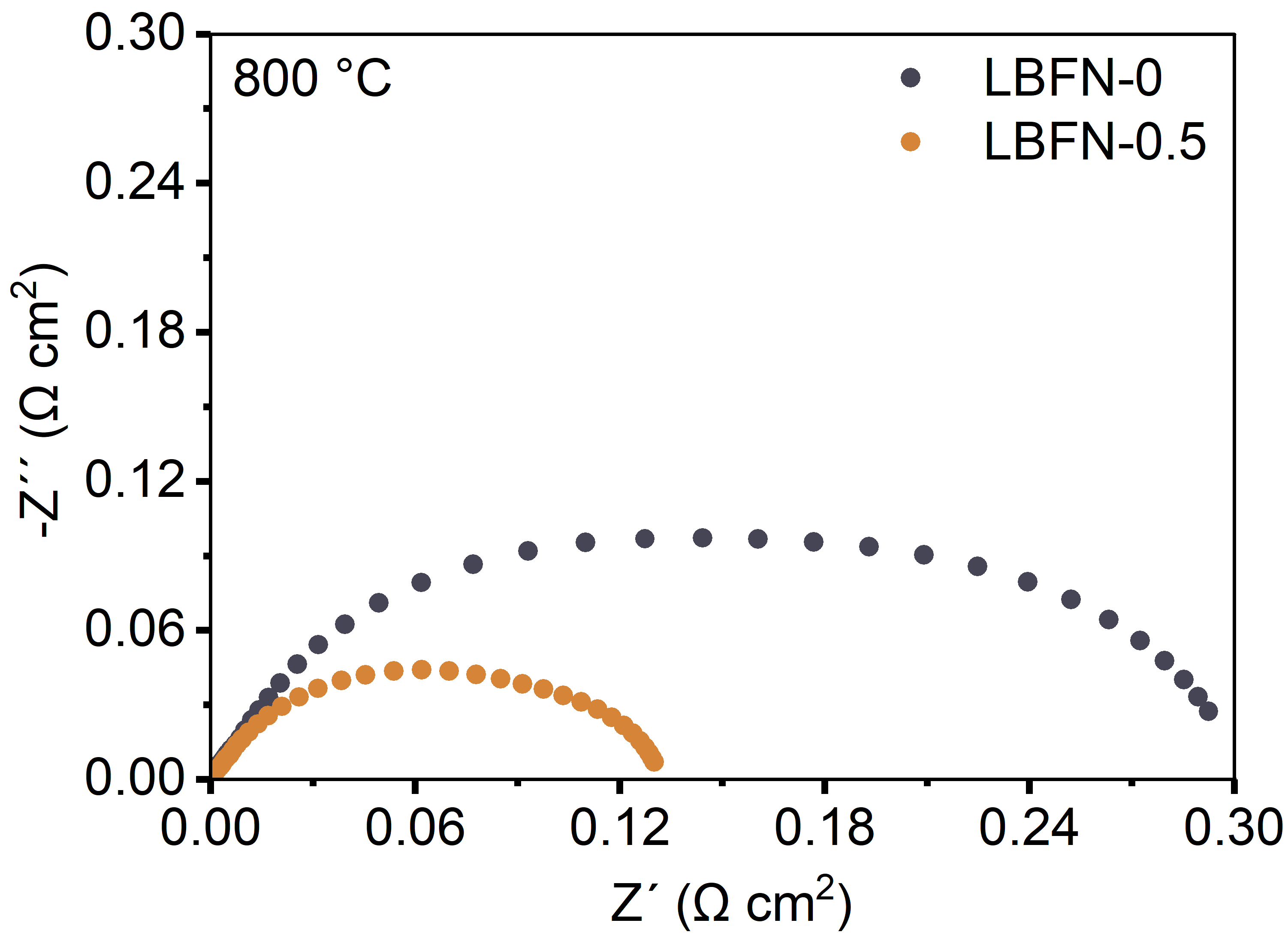
**

**Figure S14**. EIS spectra of symmetrical cells with LBFN-0 and LBFN-0.5 as anodes under H_2_ (3 vol% H_2_O) at 800 °C.


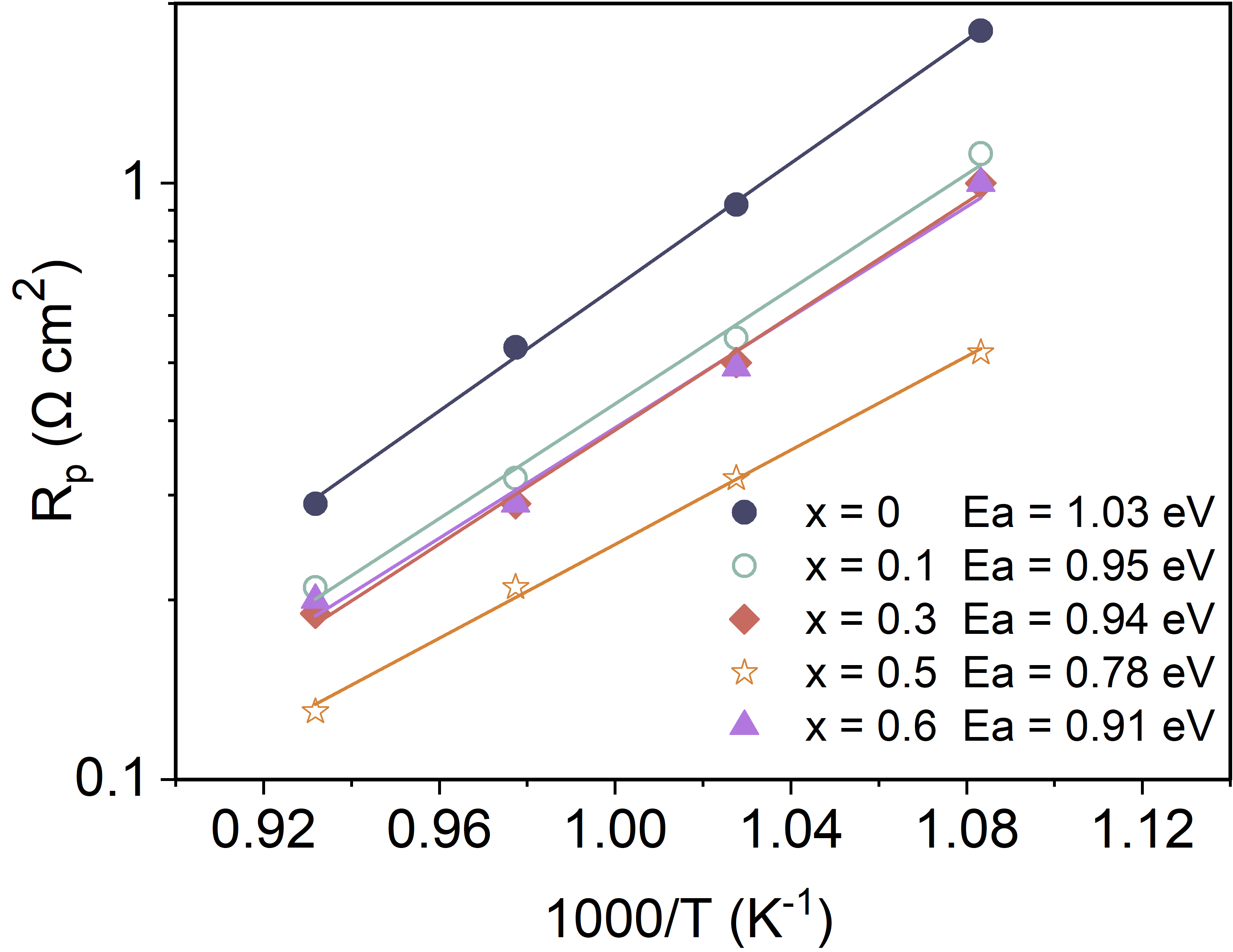


**Figure S15**. Ea analysis of La_1-x_Ba_x_Fe_0.7_Ni_0.3_O_3-δ_ (x = 0, 0.1, 0.3, 0.5, 0.6) at different temperatures under H_2_ (3 vol% H_2_O).


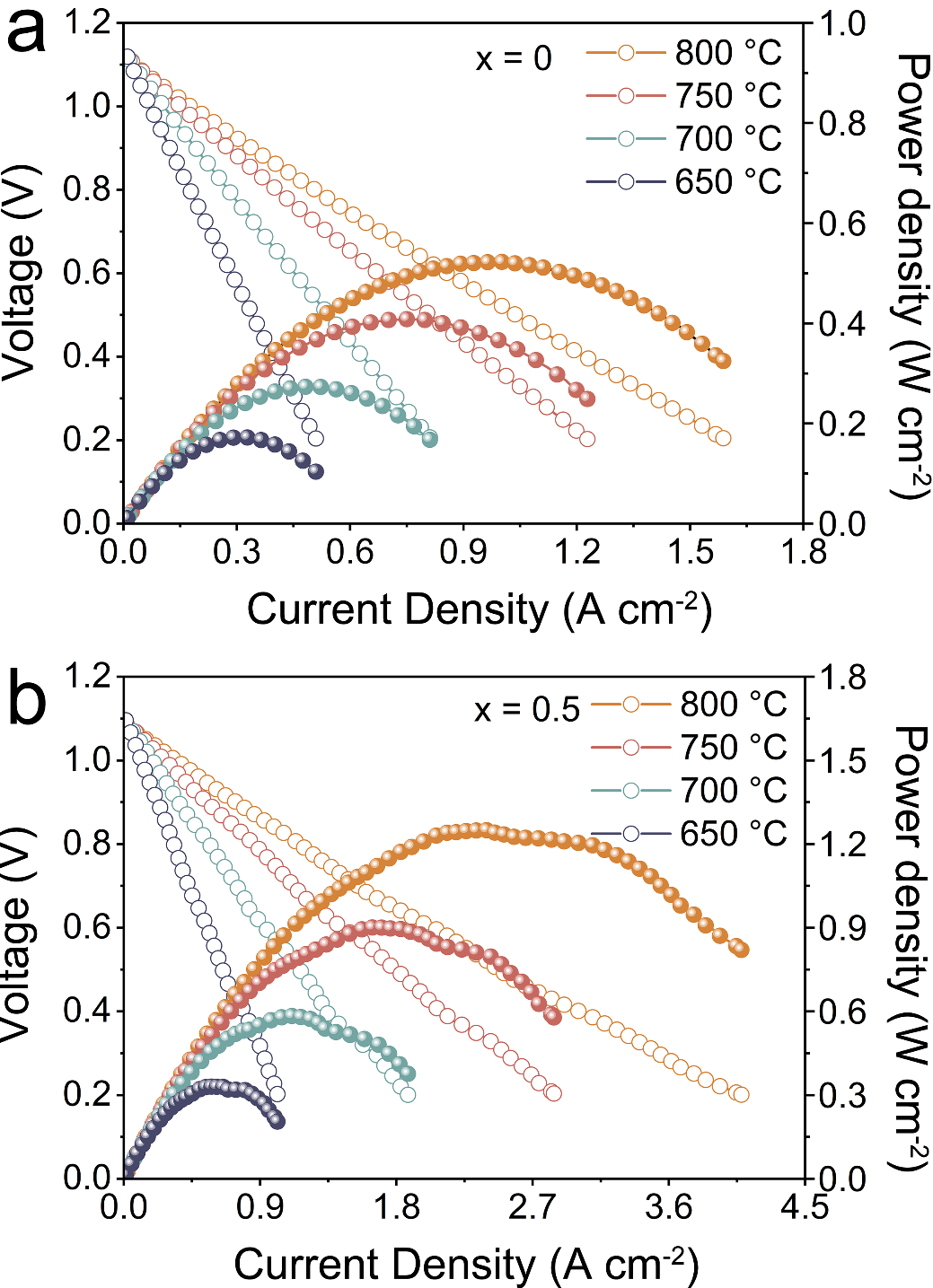


**Figure S16**. *I-V* and *I-P* curves of LSGM-supported single cells with LBFN-0 and LBFN-0.5 anode at 800 °C under H_2_ (3 vol% H_2_O).


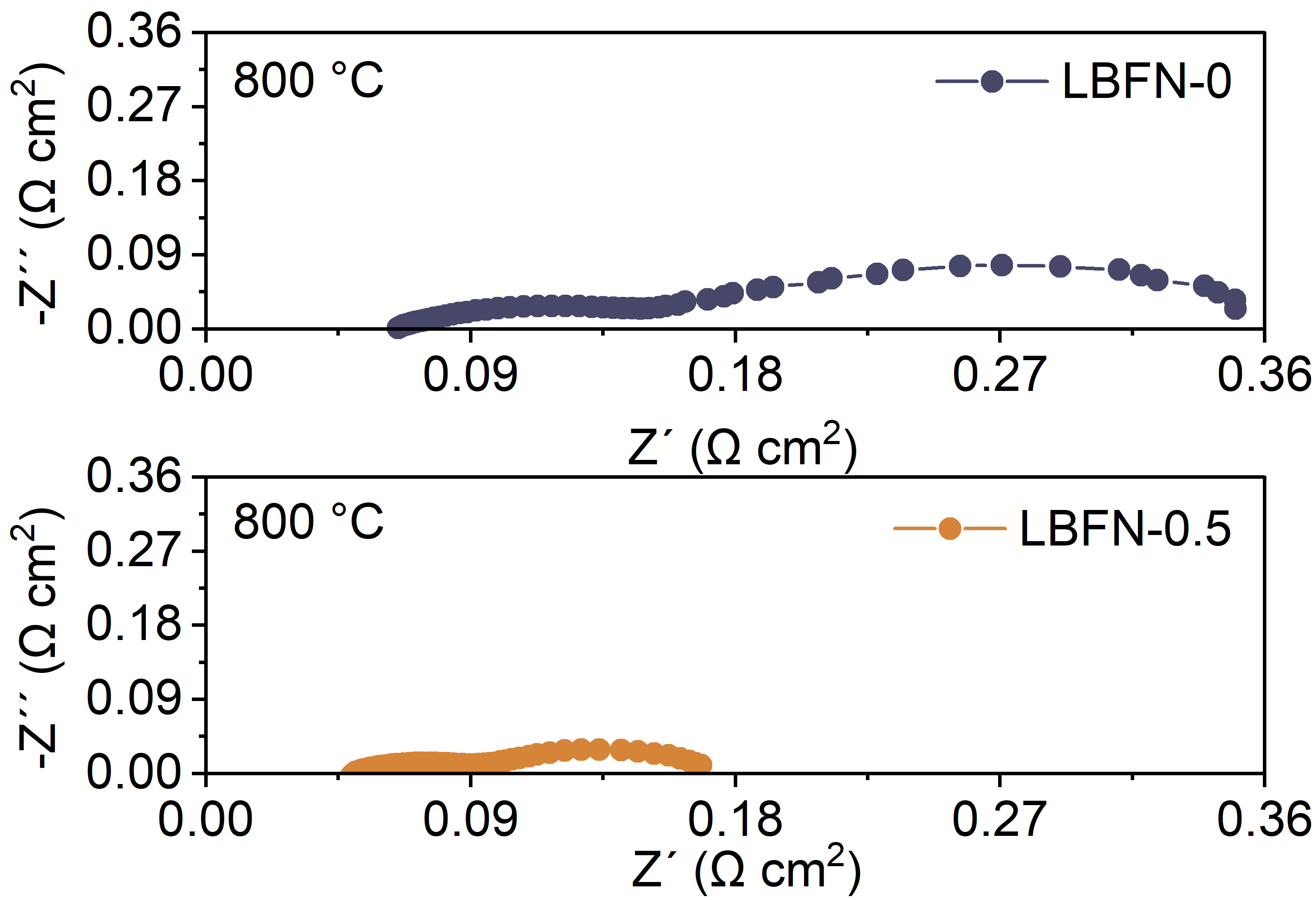


**Figure S17**. EIS spectra of the single cell with LBFN-0 and LBFN-0.5 anode under H_2_ (3 vol% H_2_O) at 800 °C.


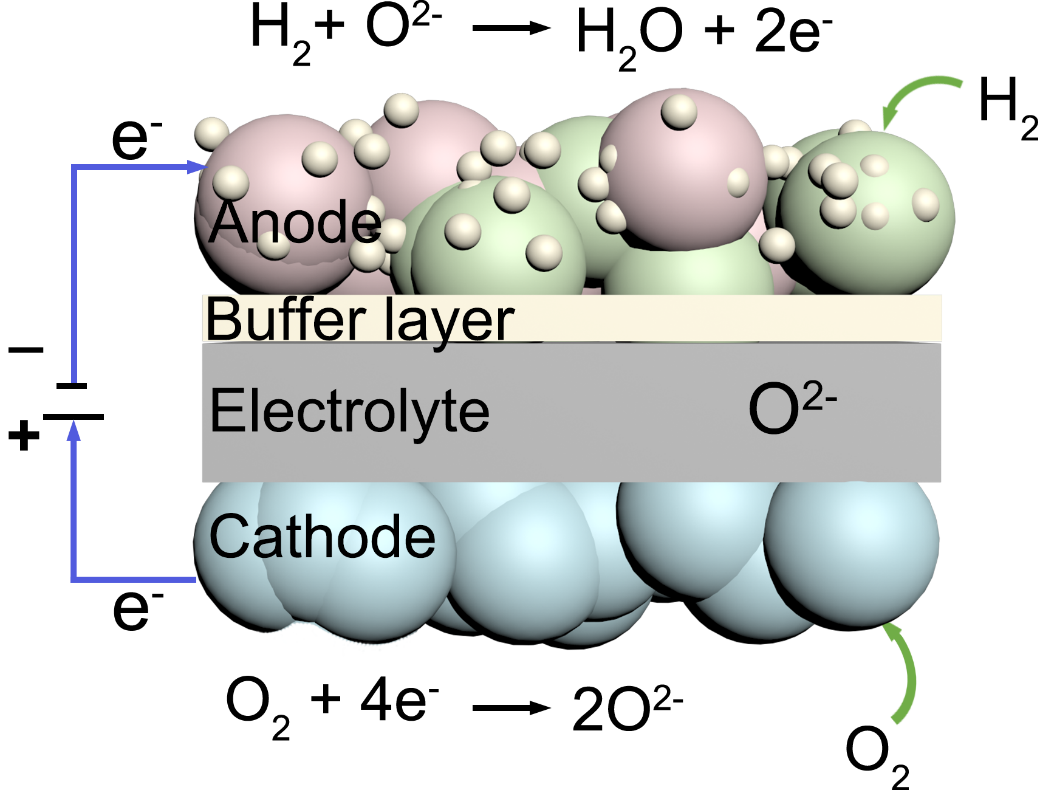


**Figure S18**. Schematic diagram of electrolyte-supported single cell where LBFN-x, LDC, LSGM, and LSCF work as the anode, buffer layer, electrolyte, and cathode, respectively.

**
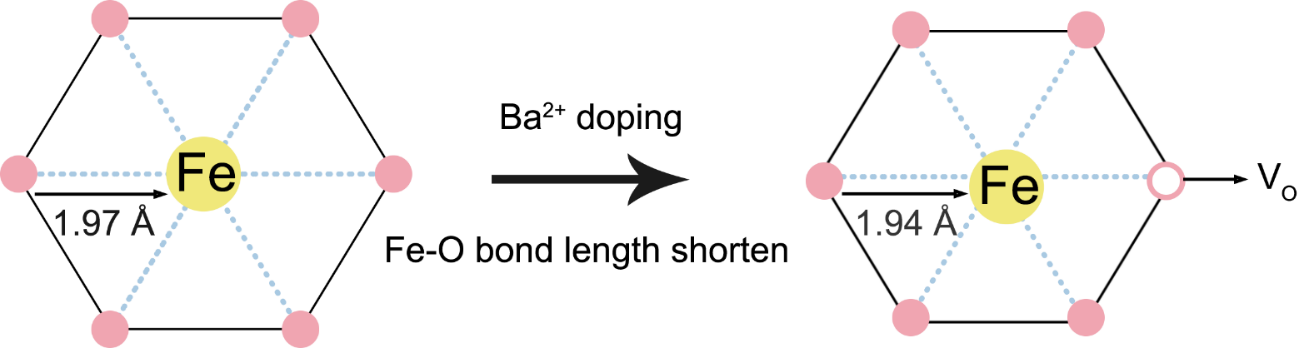
**

**Figure S19**. Schematic diagram illustrating the Fe-O bonds become shorter and the coordination number decreases.


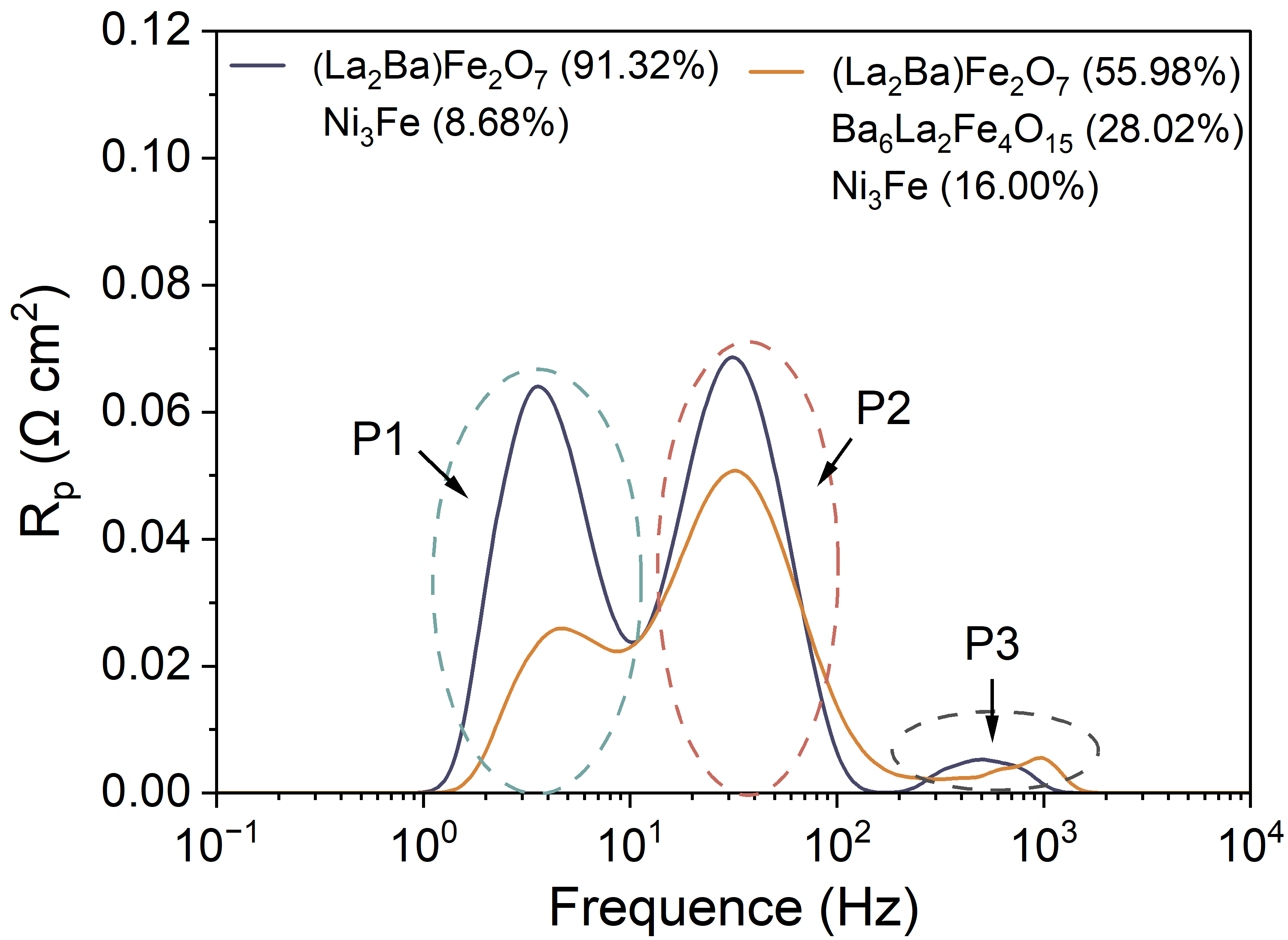


**Figure S20**. DRT analyses of (La_2_Ba)Fe_2_O_7_ (91.32%)-Ni_3_Fe (8.68%), namely LBFN-0.3, and (La_2_Ba)Fe_2_O_7_ (55.98%)-Ba_6_La_2_Fe_4_O_15_ (28.02%)-Ni_3_Fe (16.00%), namely LBFN-0.5, with different phase ratios.

In the DRT analysis of symmetrical cells with different phase ratios due to the appearance of 28.02% Ba_6_La_2_Fe_4_O_15_ in LBFN-0.5, the peak intensity in the low-frequency region (P1) is significantly reduced, indicating that the unique structures of (La_2_Ba)Fe_2_O_7_ and Ba_6_La_2_Fe_4_O_15_ (with larger interstitial spaces) favorites rapid O^2-^ transport and accelerate gas diffusion. The decrease in the intermediate-frequency region (P2) is attributed to the presence of additional catalytic sites (FeO_4_) in Ba_6_La_2_Fe_4_O_15_ and the increased content of Ni_3_Fe nanoparticles with high catalytic activity (from 8.68% in LBFN-0.3 to 16.00% in LBFN-0.5), which promotes H_2_ adsorption and dissociation.


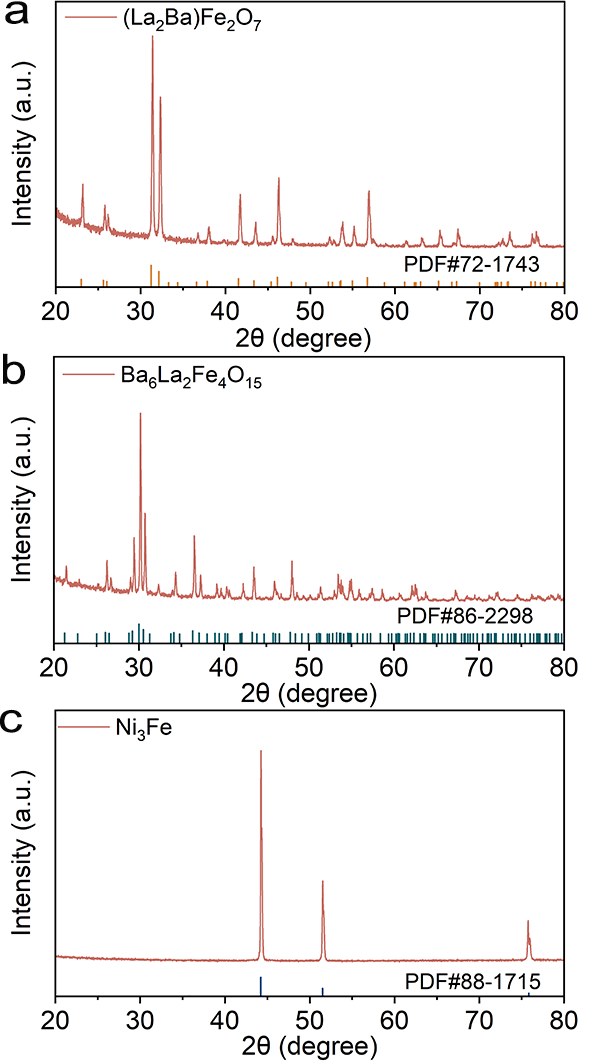


**Figure S21**. XRD patterns of different phases: a) (La_2_Ba)Fe_2_O_7_, b) Ba_6_La_2_Fe_4_O_15_, and c) Ni_3_Fe.

We have synthesized the three individual phases, namely (La_2_Ba)Fe_2_O_7_, Ba_6_La_2_Fe_4_O_15_, and Ni_3_Fe, as reference samples. XRD data confirms the phase formation and the peaks agree well with the corresponding PDF standards card.


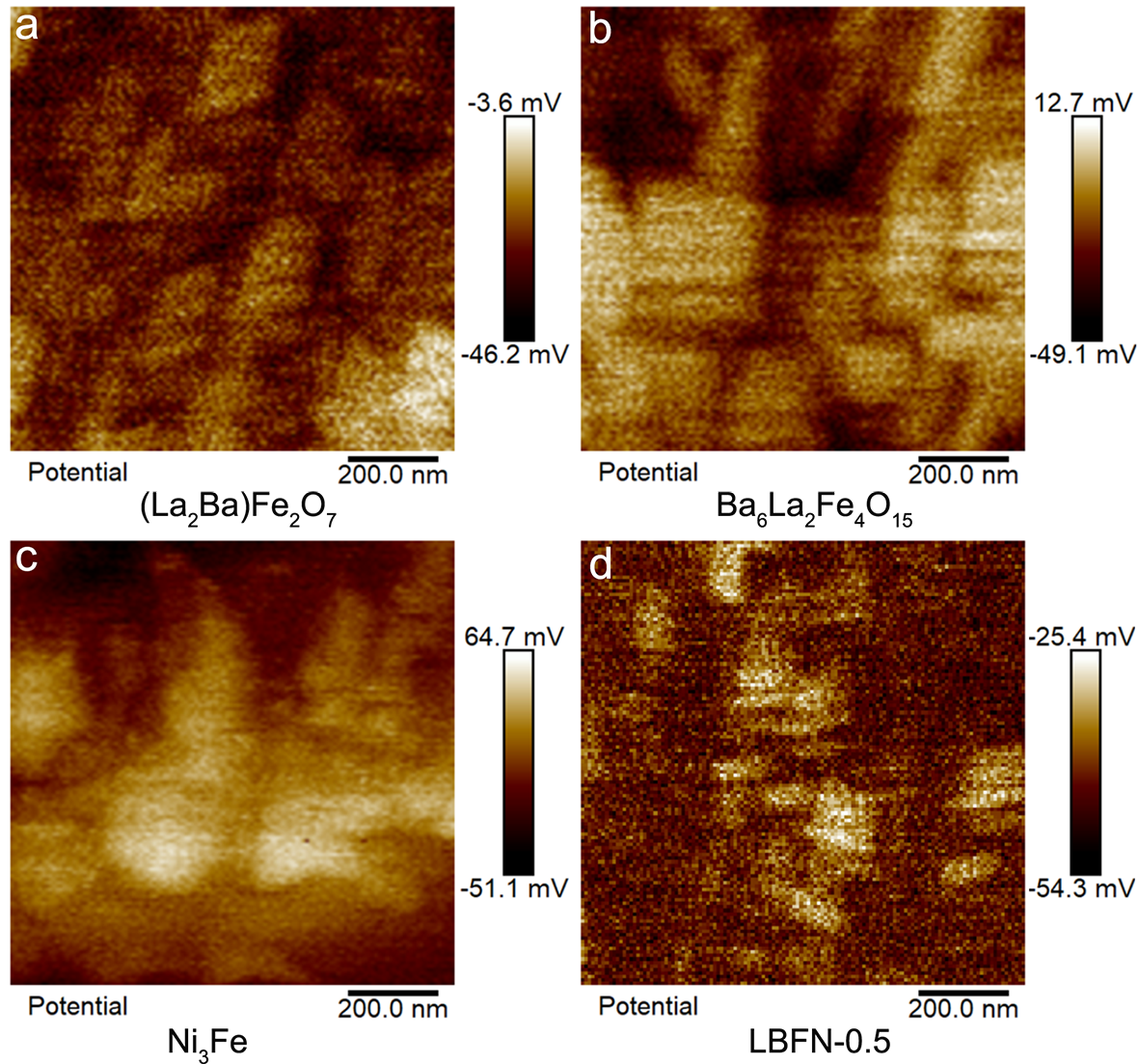


**Figure S22**. Surface potential measurement of a) (La_2_Ba)Fe_2_O_7_, b) Ba_6_La_2_Fe_4_O_15_, c) Ni_3_Fe, and d) LBFN-0.5 after reduction under H_2_ (3 vol% H_2_O) at 800 °C for 3 h.

Using KPFM, we have measured and calculated the work functions: 4.66 eV for (La_2_Ba)Fe_2_O_7_, 4.68 eV for Ba_6_La_2_Fe_4_O_15_, 4.69 eV for Ni_3_Fe, and 4.65 eV for LBFN-0.5.


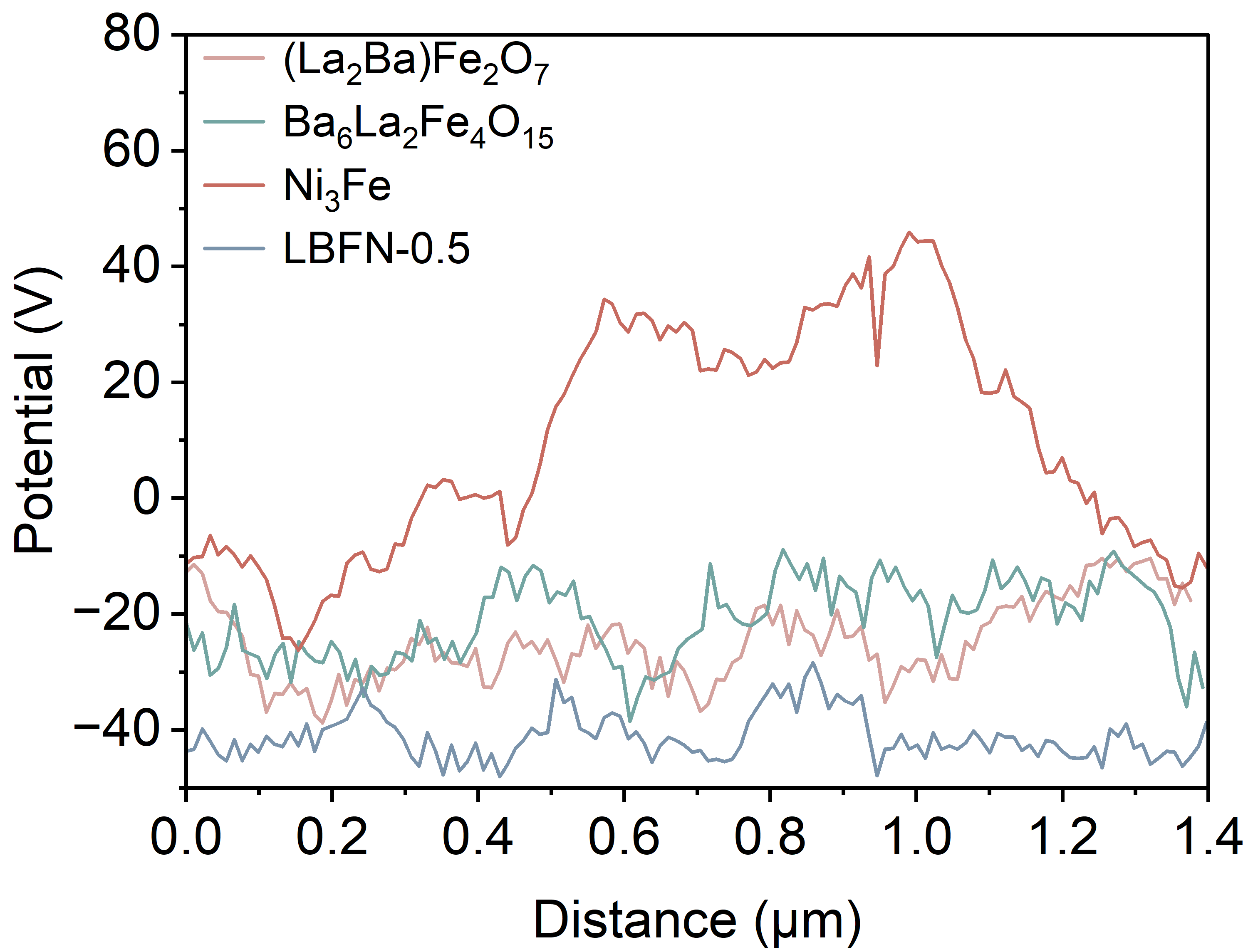


**Figure S23**. Comparison of surface potential curves for different phase structures.

**Table S1**. Crystallographic details of reduced La_1-x_Ba_x_Fe_0.7_Ni_0.3_O_3-δ_ (x = 0, 0.1, 0.3, 0.5, 0.6) conducted by Rietveld refinement of XRD data.

| Samples | Crystal parameters | Lattice parameters | Phase proportions | Refinement parameters |
| --- | --- | --- | --- | --- |
| LBFN-0 | LaFeO_3_ | a = 5.55 Å b = 5.56 Å c = 7.86 Å  α = β = γ = 90° | 75.34% | Rp = 5.81  Rwp = 7.35  GOF = 1.52 |
|  | Ni_3_Fe | a = b = c = 3.55 Å  α = β = γ = 90° | 5.79% |  |
|  | La_2_O_3_ | a = b = 3.93 Å c = 6.14 Å  α = β = 90° γ = 120° | 18.87% |  |
| LBFN-0.1 | LaFeO_3_ | a = b = c = 3.93Å  α = β = γ = 90° | 79.75% | Rp = 7.49  Rwp = 9.68  GOF = 10.5 |
|  | (La_2_Ba)Fe_2_O_7_ | a = b =3.92 Å c =20.77 Å  α = β = γ = 90° | 16.22% |  |
|  | Ni_3_Fe | a = b = c = 3.51 Å  α = β = γ = 90° | 4.03% |  |
| LBFN-0.3 | (La_2_Ba)Fe_2_O_7_ | a = b =3.93 Å c =20.79 Å  α = β = γ = 90° | 91.32% | Rp = 6.65  Rwp = 8.34  GOF = 1.69 |
|  | Ni_3_Fe | a = b = c = 3.55 Å  α = β = γ = 90° | 8.68% |  |
| LBFN-0.5 | (La_2_Ba)Fe_2_O_7_ | a = b =3.93 Å c =20.81 Å  α = β = γ = 90° | 55.98% | Rp = 7.25  Rwp = 9.57  GOF = 1.48 |
|  | Ba_6_La_2_Fe_4_O_15_ | a = b =11.86 Å c =7.13 Å  α = β = 90° γ = 120° | 28.02% |  |
|  | Ni_3_Fe | a = b = c = 3.56 Å  α = β = γ = 90° | 16.00% |  |
| LBFN-0.6 | (La_2_Ba)Fe_2_O_7_ | a = b =3.93 Å c =20.83 Å  α = β = γ = 90° | 27.65% | Rp = 7.15  Rwp = 9.02  GOF = 3.33 |
|  | Ba_6_La_2_Fe_4_O_15_ | a = b =11.85 Å c =7.11 Å  α = β = 90° γ = 120° | 66.98% |  |
|  | Ni_3_Fe | a = b = c = 3.57 Å  α = β = γ = 90° | 5.37% |  |

**Table S2**. XPS fitting results of Fe for LBFN-0 and LBFN-0.5 before and after reduction with H_2_ (3 vol% H_2_O).

| Samples | Fe^2+^ | Fe^3+^ | Fe^0^ | Avarage valence |
| --- | --- | --- | --- | --- |
| LBFN-0 | 70.37 | 29.63 | - | 2.29 |
| LBFN-0.5 | 75.61 | 24.39 | - | 2.24 |
| R-LBFN-0 | 76.36 | 19.95 | 3.69 | 2.13 |
| R-LBFN-0.5 | 78.54 | 17.73 | 3.57 | 2.10 |

**Table S3**. XANES fitting results of LBFN-0 and LBFN-0.5.

| Sample | Shell | N^a^ | R^b^ (Å) | σ^2c^ (Å^2^) | ΔE_0_^d^ (eV) | R factor^e^ |
| --- | --- | --- | --- | --- | --- | --- |
| LBFN-0 | Fe-O | 6.02 | 1.97 | 0.01014 | -6.96 | 0.0058 |
| LBFN-0.5 | Fe-O | 5.09 | 1.94 | 0.00998 | -5.89 | 0.0080 |

^a)^ Coordination numbers. ^b)^ Bond distance. ^c)^ Debye-Waller factors. ^d)^ Inner potential correction. ^e)^ Goodness of fit. *Ѕ*_0_^2^ was set to 0.76, according to the experimental EXAFS fit of Fe_2_O_3_ by fixing *N* as the known crystallographic value. The data range used for data fitting in k-space (∆k) and R-space (∆R) are 3-12 Å^-1^ and 1-2.1 Å, respectively.

**Table S4**. Summary of PPD values for Fe-based double perovskite anode materials in H_2_ with 3 vol% H_2_O.

| Electrode | Electrolyte & thickness (μm) | PPD (W cm^-2^) | Ref. |
| --- | --- | --- | --- |
| Pr_0.4_Sr_0.6_Co_0.2_Fe_0.7_Nb_0.1_O_3-δ_ | LSGM 300 | [0.93@800](mailto:0.93@800) °C | [1] |
| Pr_0.8_Sr_1.2_(Co,Fe)_0.8_Nb_0.2_O_4+δ_ –CFA | LSGM 300 | [0.78@800](mailto:0.78@800) °C | [2] |
| (Pr_0.5_Sr_0.5_)_0.9_Fe_0.8_Ru_0.1_Nb_0.1_O_3-δ_ | LSGM 300 | [0.683@800](mailto:0.683@800) °C | [3] |
| Sr_1.95_Fe_1.4_Co_0.1_Mo_0.5_O_6-δ_ | LSGM 300 | [1.01@800](mailto:1.01@800) °C | [4] |
| Sr_0.95_Ti_0.3_Fe_0.63_Ni_0.07_O_3-δ_ | LSGM 300 | [0.95@800](mailto:0.95@800) °C | [5] |
| Sr_2_FeMo_0.65_Ni_0.35_O_6−δ_ | LSGM 300 | [0.79@800](mailto:0.79@800) °C | [6] |
| Pr_0.5_Sr_0.5_C_r0.1_Fe_0.7_Ni_0.2_O_3−δ_ | LSGM 240 | 0.62@800 °C | [7] |
| La_0.5_Ba_0.5_Mn_0.8_Fe_0.1_Co_0.1_O_3−δ_ | LSGM 300 | 0.97@800 °C | [8] |
| La_0.6_Sr_0.4_Mn_0.2_Fe_0.8_O_3−δ_-GDC | LSGM 280 | 0.64@800 °C | [9] |
| Sr_2_FeMo_2/3_Mg_1/3_O_6-δ_ | LSGM 300 | 0.8@800 °C | [10] |
| Ru@Ru-Sr_2_Fe_1.5_Mo_0.5_O_6-δ_(SFM)/Ru-Gd_0.1_Ce_0.9_O_2-δ_(GDC) | LSGM 230 | [1.03@800](mailto:1.03@800) ℃ | [11] |
| La_0.5_Ba_0.5_Fe_0.7_Ni_0.3_O_3_ | LSGM 250 | [1.25@800](mailto:1.25@800) °C | This work |

**References**

1. C. Yang, J. Li, Y. Lin, J. Liu, F. Chen, and M. Liu, “In Situ Fabrication of CoFe Alloy Nanoparticles Structured (Pr_0.4_Sr_0.6_)_3_(Fe_0.85_Nb_0.15_)_2_O_7_ Ceramic Anode for Direct Hydrocarbon Solid Oxide Fuel Cells,” *Nano Energy* 11 (2015): 704-710, https://doi.org/10.1016/j.nanoen.2014.12.001.

2. C. Yang, Z. Yang, C. Jin, G. Xiao, F. Chen, and M. Han, “Sulfur-Tolerant Redox-Reversible Anode Material for Direct Hydrocarbon Solid Oxide Fuel Cells,” *Advanced Materials* 24 (2012): 1439-1443, https://doi.org/10.1002/adma.201104852.

3. M. Qin, Y. Xiao, H. Yang, et al., “Ru/Nb Co-Doped Perovskite Anode: Achieving Good Coking Resistance in Hydrocarbon Fuels via Core-Shell Nanocatalysts Exsolution,” *Applied Catalysis B: Environmental* 299 (2021): 120613, https://doi.org/10.1016/j.apcatb.2021.120613.

4. C. Xu, W. Sun, R. Ren, et al., “A Highly Active and Carbon-Tolerant Anode Decorated with in Situ Grown Cobalt Nano-Catalyst for Intermediate-Temperature Solid Oxide Fuel Cells,” *Applied Catalysis B: Environmental* 282 (2021): 119553, https://doi.org/10.1016/j.apcatb.2020.119553.

5. T. Zhu, H. E. Troiani, L. V. Mogni, M. Han, and S. A. Barnett, “Ni-Substituted Sr(Ti,Fe)O_3_ SOFC Anodes: Achieving High Performance via Metal Alloy Nanoparticle Exsolution,” *Joule* 2 (2018): 478-496, https://doi.org/10.1016/j.joule.2018.02.006.

6. Z. Du, H. Zhao, S. Yi, et al., “High-Performance Anode Material Sr_2_FeMo_0.65_Ni_0.35_O_6-δ_ with In Situ Exsolved Nanoparticle Catalyst,” *ACS Nano* 10 (2016): 8660–8669, https://doi.org/10.1021/acsnano.6b03979.

7. J. Yan, H. Chen, Y. W. Li, S.-D. Li, and Z. Shao, “Bifunctional Electrocatalysts Pr_0.5_Sr_0.5_Cr_0.1_Fe_0.9-x_Ni_x_O_3-δ_ (x = 0.1, 0.2) for the HOR and ORR of a Symmetric Solid Oxide Fuel Cell,” *Journal of Materials Chemistry A* 11 (2023): 21839-21845, https://doi.org/10.1039/D3TA03475E.

8. N. Hou, T. Yao, P. Li, et al., “A-Site Ordered Double Perovskite with in Situ Exsolved Core–Shell Nanoparticles as Anode for Solid Oxide Fuel Cells,” *ACS Applied Materials & Interfaces* 11 (2019): 6995-7005, https://doi.org/10.1021/acsami.8b19928.

9. Y. S. Chung, T. Kim, T. H. Shin, et al., “In Situ Preparation of a La_1.2_Sr_0.8_Mn_0.4_Fe_0.6_O_4_ Ruddlesden-Popper Phase with Exsolved Fe Nanoparticles as an Anode for SOFCs,” *Journal of Materials Chemistry A* 5 (2017): 6437-6446, https://doi.org/10.1039/C6TA09692A.

10. Z. Du, H. Zhao, S. Li, et al., “Exceptionally High Performance Anode Material Based on Lattice Structure Decorated Double Perovskite Sr_2_FeMo_2/3_Mg_1/3_O_6-δ_ for Solid Oxide Fuel Cells,” *Advanced Energy Materials* 8 (2018): 1800062, https://doi.org/10.1002/aenm.201800062.

11. F. Hu, K. Chen, Y. Ling, et al., “Smart Dual-Exsolved Self-Assembled Anode Enables Efficient and Robust Methane-Fueled Solid Oxide Fuel Cells,” *Advanced Science* 11 (2024): 2306845, https://doi.org/10.1002/advs.202306845.
